# Synthesis and activity of a novel inhibitor of nonsense-mediated mRNA decay†
†Electronic supplementary information (ESI) available: Experimental procedures and spectroscopic data. See DOI: 10.1039/c5ob02482j



**DOI:** 10.1039/c5ob02482j

**Published:** 2016-01-07

**Authors:** Victoria J. B. Gotham, Melanie C. Hobbs, Ryan Burgin, David Turton, Carl Smythe, Iain Coldham

**Affiliations:** a Department of Chemistry , University of Sheffield , Sheffield , S3 7HF , UK . Email: i.coldham@sheffield.ac.uk; b Department of Biomedical Science , University of Sheffield , Sheffield , S10 2TN , UK

## Abstract

During efforts to prepare the known compound **NMDI1**, a new tetracyclic compound, called **VG1**, was prepared in six steps. This compound was found to have good activity as an inhibitor of nonsense-mediated mRNA decay.

## Abstract 2

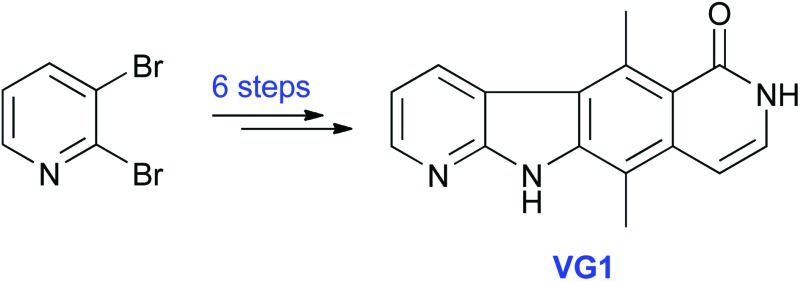
A new route to a tetracyclic lactam was developed and the product, called **VG1**, was found to inhibit nonsense-mediated mRNA decay at μM concentrations.

A significant proportion of genetic diseases, as well as many cancers, are associated with a particular type of mutation which gives rise to a premature termination codon (PTC) before the genuine stop codon.^1^ PTC-associated diseases include cases of Duchenne muscular dystrophy, cystic fibrosis, β-thalassaemia, retinitis pigmentosa, von Willebrand disease, Robinow syndrome, haemophilia, spinal muscular atrophy and Rett syndrome. mRNAs containing PTCs are detected and degraded by a process known as nonsense-mediated mRNA decay (NMD),^2–4^ which reduces the expression of the corresponding gene to a low level. It is the subsequent lack of protein that causes the symptoms of PTC-associated diseases. The PTCs that trigger NMD can occur due to single base pair mutations, reading frame shifts due to deletions or insertions, or mutations in splice sites or splicing regulatory sequences that give rise to aberrant splicing.^2–4^ At the mRNA level, PTCs can arise from errors in transcription, transcription initiation upstream of the correct site coupled with the location of a termination codon in the region before the proper start site, alternative splicing, or programmed frameshifts during translation.^2–4^

One potential approach to the treatment of diseases caused by PTC mutations is the inhibition of NMD. Therefore there is a need to discover and develop drugs that inhibit NMD selectively. In addition, NMD inhibitors can be useful tools for studying the NMD mechanism and related pathways that utilise some of the same factors. In 2007, Lejeune and co-workers^5^ found that a tetracyclic compound called **NMDI1** (Fig. 1) was able to inhibit both nucleus-associated and cytoplasmic NMD. The compound appears to be a specific inhibitor of NMD: it does not affect the splicing of pre-mRNA reporter transcripts, the amount of pre-mRNA, translation efficiency or the miRNA decay pathway.^5^ It is non-cytotoxic, even at high concentrations, and does not induce the formation of stress granules.^5^

**Fig. 1:**
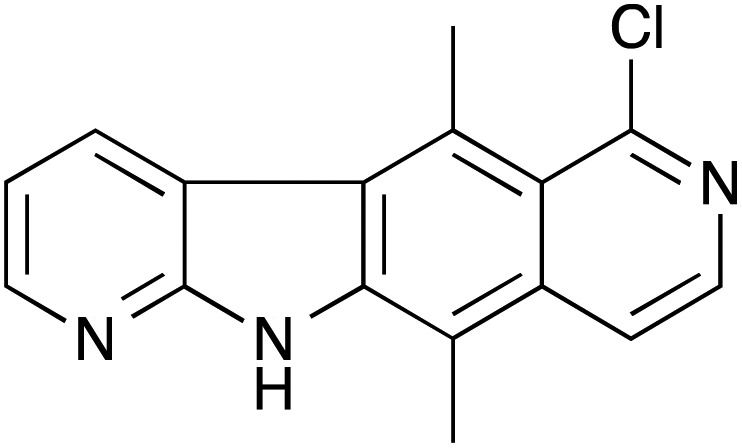
Structure of NMD inhibitor **NMDI1**.

The synthesis of **NMDI1** was reported by Bisagni and co-workers.^6,7^ The overall yield was poor (0.5% over 11 steps) and we had difficulties on attempted repetition of the published procedure (see ESI†). Keeling and co-workers have recently reported a synthesis of **NMDI1** by a modified route (total 13 steps) in which the final 7 steps were identical to those used by Bisagni and co-workers; however, they report no experimental details.^8^

There is clearly a need for a NMD inhibitor whose synthesis is more efficient and reproducible. We have synthesised in only 6 steps a close structural analogue of **NMDI1**, called **VG1**, by using a different route from that described above.^6–8^ We found that **VG1** inhibits NMD equally well as **NMDI1** and is therefore a suitable candidate for use as a tool to investigate NMD and related mechanisms, and possibly for development as a drug for diseases caused by PTC mutations.

**VG1** was synthesised from commercially available 2,3-dibromopyridine and 2,5-dimethylaniline (Scheme 1). These were heated at 140 °C for 16 h to give bromide **1** in 76% yield. An aldehyde was then installed *para* to the amino group. The use of POCl_3_ and DMF (Vilsmeier reaction)^9,10^ failed to give the desired aldehyde, but the use of AlCl_3_ and MeOCHCl_2_ (Rieche formylation)^11,12^ was successful and gave aldehyde **2**. This compound was not purified but was treated directly with NaClO_2_ and NH_2_SO_3_H as a chlorine scavenger,^13–15^ to give the carboxylic acid **3** in 66% yield from bromide **1**. The acid was converted to amide **4** in 94% yield by using the commercially available aminoacetaldehyde diethylacetal and the coupling reagent COMU.^16^ Deprotection of the acetal with polyphosphoric acid was followed by *in situ* cyclisation and dehydration^17^ to give compound **5** in 70% yield. An intramolecular palladium-catalysed cross-coupling reaction^18^ was used to convert bromide **5** into tetracycle **VG1** in 54% yield.

**Scheme 1:**
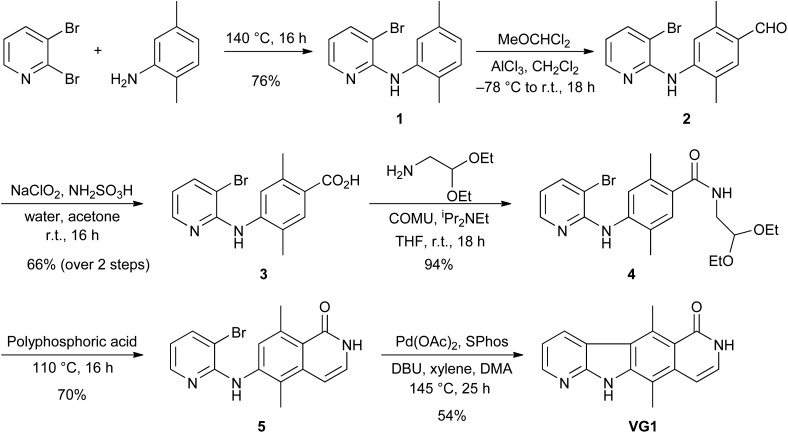
Synthesis of **VG1**.

Attempted conversion of the lactam **5** to the chloropyridine was unsuccessful by using POCl_3_, PhP(O)Cl_2_, PCl_3_, or PCl_5_ in POCl_3_. Chlorination of **VG1** was also attempted but the only isolable products obtained, after treatment of either compound **5** or **VG1** with PCl_5_ in POCl_3_, were *N*-chloro derivatives (see ESI†). Chlorination on the nitrogen atom of an amide has literature precedent.^19–21^ Unfortunately, this meant that we were not able to prepare **NMDI1** using this synthesis. However, we had prepared a close analogue in **VG1** and decided to test the activity of this compound.

To test the ability of **VG1** to inhibit NMD we utilised a well-established assay for the measurement of NMD within cells.^22^ HeLa cells were transfected with either one of two reporter plasmids, pmCMV-Gl (NORM) or pmCMV-Gl (TER),^22–24^ referred to as NORM and TER. The NORM plasmid contains a hybrid human/mouse β-globin gene. This was constructed by Maquat and Kinniburgh^23^ by substituting sequences in the human β-globin gene extending from the *Bam*H1 site in exon II with analogous sequences from the mouse β-globin gene. The resulting hybrid gene contains exon I, intron I and most of exon II of the human β-globin gene, and 18 base pairs of exon II, intron II and exon III of the mouse β-globin gene. The TER plasmid contains the same chimaeric gene but with a PTC mutation (CAG to TAG) in codon 39, located in exon II. The cloning of NORM and TER sequences into the mRNA producing plasmid pCMV, and the characterisation of resultant mRNA has been previously described.^22^ TER and NORM transcripts are subject to splicing, and TER, but not NORM, spliced mRNA is subject to NMD.^22^ Fig. 2 shows a plasmid map and the structure of the hybrid genes. In order to control for transfection efficiency, cells were also transfected with the reference plasmid phCMV-MUP (referred to as MUP), which encodes mouse major urinary protein.^25^ After 24 h, the transfection medium was removed from cells and replaced with culture medium containing either DMSO or **NMDI1** or **VG1** at a final concentration of 5 μM made up from a 5 mM stock solution in DMSO. Each compound was added both to cells transfected with NORM and cells transfected with TER. After 20 h, total RNA was extracted and reverse transcribed. The amount of NORM, TER and MUP cDNA was determined by qPCR, where the amount of cDNA serves as a proxy measurement for mRNA as has been described previously.^27,28^ To ensure that the qPCR reaction products reflected the appropriately spliced mature mRNA, the cDNA generated from RNA extracted from both NORM and TER transfected cells was amplified by standard PCR using identical primers and DNA products which correspond to their cognate mRNAs were analysed for size by agarose gel electrophoresis. These showed that PCR products correspond to correctly spliced mRNA (Fig. S2 in ESI†).

**Fig. 2:**
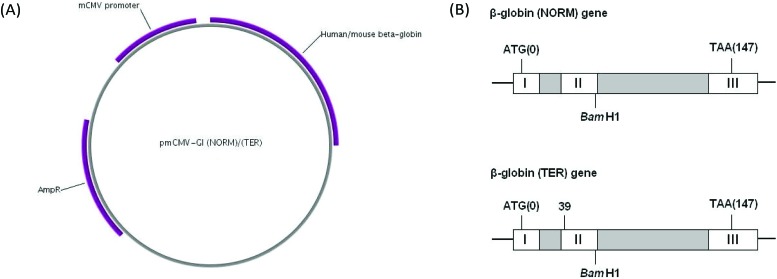
pmCMV-Gl (NORM) and pmCMV-Gl (TER) plasmids. (A) Plasmid map. The plasmids contain a mouse cytomegalovirus (mCMV) promoter, a hybrid human/mouse β-globin gene and a gene for ampicillin resistance. The map was created using the PlasMapper program.^26^ (B) Structure of the hybrid human/mouse β-globin gene. Grey boxes are introns. Start (ATG) and stop (TAA) codons are shown. The *Bam*H1 site marks the boundary between sequences from the human and mouse genes. The TER plasmid is identical to NORM, except that a PTC mutation (CAG to TAG), has been introduced in codon 39 located in exon II.

The inferred amounts of NORM and TER mRNA, each expressed as a ratio of the respective inferred amount of MUP mRNA in each sample, were compared in order to determine the NMD efficiency. As expected, for cells treated with DMSO only, the amount of TER mRNA was significantly lower than the amount of NORM mRNA (0.22 times the amount of NORM mRNA), indicating that TER mRNA was efficiently degraded by NMD (Fig. 3A, first and second bars from left). When cells were treated with **VG1**, the amount of TER mRNA increased to 0.70 times the amount of NORM mRNA, showing that NMD was inhibited (Fig. 3A, fifth and sixth bars from left). **VG1** inhibited NMD to the same extent as **NMDI1** (Fig. 3A, third and fourth bars from left).

**Fig. 3:**
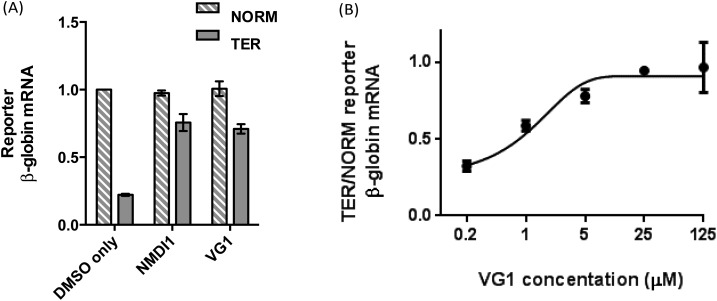
NMD inhibition by **VG1**. (A) Cells transfected with the NMD reporter constructs were treated with either DMSO or 5 μM **NMDI1** or **VG1** for 20 h. The amount of reporter β-globin mRNA (NORM or TER) is controlled to the amount of MUP mRNA. The data are normalised to the amount of NORM mRNA (controlled to the amount of MUP mRNA) for DMSO-treated cells. Error bars show the standard error in the mean obtained from three independent experiments. (B) Cells transfected with the NMD reporter constructs were treated with **VG1** at the concentrations indicated for 20 h. The amount of TER β-globin mRNA is expressed relative to the amount of NORM β-globin mRNA (both controlled to the amount of MUP mRNA) for each concentration. Error bars show the standard error in the mean obtained from three independent experiments for 0.2, 1 and 5 μM **VG1** and from two independent experiments for 25 and 125 μM **VG1**.

The effect of the dose of **VG1** on NMD inhibition was then investigated. Cells were transfected with the NMD reporter plasmids, as described above. After 24 h, the transfection medium was removed from cells and replaced with culture medium containing either DMSO or **VG1** at a final concentration of 0.2, 1, 5, 25 or 125 μM made up from a stock solution in DMSO. After 20 h, total RNA was extracted and reverse transcribed. The amount of NORM, TER and MUP cDNA was determined by qPCR and the inferred amounts of NORM and TER mRNA, each expressed as a ratio of the respective inferred amount of MUP mRNA in each sample, were compared in order to determine the NMD efficiency. To ensure that the PCR primers used generated a reaction product corresponding to spliced RNA and not unspliced pre-mRNA, the amplified PCR products from RNA extracted from cells transfected with either NORM, or TER, were examined by DNA agarose gel electrophoresis (Fig. S2†). In each case, these showed the presence of a DNA fragment of 150 bp corresponding to correctly spliced mRNA. In qPCR analyses, the amount of TER mRNA relative to NORM mRNA, and therefore NMD inhibition, increased as **VG1** concentration increased, reaching a maximum at 25 μM, at which concentration the amount of TER mRNA was 0.94 times the amount of NORM mRNA (Fig. 3B). There was no further NMD inhibition at 125 μM. Thus high levels of inhibition of NMD were achieved by treating cells with 5–25 μM **VG1**, assuming that if NMD were fully inhibited, the amount of TER mRNA would be equal to the amount of NORM mRNA.

NMDI1 has been reported to have little effect on cell viability.^5^ When cells were seeded as normal (10^4^ cells per cm^2^) and treated with 5 μM **VG1** for 24 h, the rate of proliferation appeared similar to cells treated with DMSO alone (Fig. S1A in ESI†) and there was little evidence of increased pyknosis or karyorrhexis (indicators of necrosis or late-stage apoptosis) suggesting that, as with **NMDI1**, effective doses on this timescale are not toxic for cell survival. The effect of **VG1** dose on cells after 20 h exposure was also investigated (Fig. S1B†). As before, at concentrations up to 5 μM, there was no obvious effect on cell number or morphology. When **VG1** was added to cell media at 25 μM and cells were incubated with compound for 20 h, there was some indication of altered cell morphology in a minority of cells, suggestive of some toxicity at higher concentrations and possible induction of apoptosis (Fig. S1B†). Further analyses of the effects of longer exposure at 25 μM or the use of higher concentrations were not possible due to insolubility of **VG1** in cell media at high concentrations. Exploration of the induction of apoptosis at an intermediate concentration of **VG1** (10 μM, Fig. S1C†) was evaluated by immunoblotting total cell lysates for the appearance of the cell marker of apoptotic induction, activated (cleaved) Caspase 3.^29^ These data (Fig. S1C†) indicated that, at this concentration and time scale, there was no evidence of elevated levels of apoptotic cell death under these conditions. Taken together our data suggest that, as with **NMDI1**, effective doses of **VG1** over a 24 h time scale are not toxic for cell survival.

Inhibiting NMD is a promising approach towards treating diseases caused by PTC mutations as, in many cases, the truncated protein that would result if the PTC-containing mRNA were to be translated could be at least partially functional and could relieve some of the symptoms of the disease. Obviously, the function of the truncated protein would depend on the location of the PTC within the mRNA. As an example of the potential of this approach, when cultured fibroblasts from a patient with Ullrich's disease (which is caused by a PTC in the gene for the extracellular matrix protein collagen VI α2) were treated with inhibitors of the NMD factor SMG1, there was an increase in the expression of truncated collagen VI α2 which partially restored the function of the extracellular matrix.^30^ However, SMG1 inhibitors are unlikely to be able to be used clinically as they are toxic and are not specific for SMG1. Other small molecules that have been used to inhibit NMD have not yet reached clinical trials of NMD inhibition.^31–36^

One approach for treating PTC-associated diseases involves the use of a class of compounds called ribosome readthrough promoters.^37,38^ These compounds induce the ribosome to “read through” a PTC, allowing a full-length protein to be produced from the transcript. However, the efficacy of readthrough promoters is limited by a paucity of PTC-containing mRNAs available for readthrough, as most are degraded rapidly by NMD. Inhibiting NMD, in combination with readthrough therapy, should mean that fewer PTC-containing mRNAs are degraded and therefore more are available for readthrough. This approach has been used by Lejeune and co-workers,^33^ who found that the effect of 5 μM NMD inhibitor amlexanox combined with 25 μM readthrough promoter **PTC124** was greater than that of either drug on its own in increasing iodide efflux in 6CFSMEo cystic fibrosis cells. Keeling and co-workers have shown that **NMDI1** was able to enhance the therapeutic effect of at least a subset of readthrough promoters called aminoglycosides in Idua^W392X^ mice (which have a premature stop codon in the gene encoding α-l-iduronidase).^8^

In this paper, we have developed a synthesis of an analogue of **NMDI1** and have found that our compound, **VG1**, inhibited NMD to the same extent as **NMDI1**. Our synthesis requires only 6 steps and proceeds in 18% overall yield. Lejeune and co-workers presented evidence that **NMDI1** inhibits the interaction between two key NMD factors, UPF1 and SMG5.^5^ UPF1 forms part of a complex that recognises the PTC-containing mRNA after the first round of translation terminates at the PTC. The protein undergoes cycles of phosphorylation and de-phosphorylation, which allow it to take part in multiple rounds of NMD. SMG5 is one of the proteins that mediates the de-phosphorylation of UPF1, and inhibition of the UPF1-SMG5 interaction may cause UPF1 to become trapped in a hyper-phosphorylated state. Given the similarity between the structures of **VG1** and **NMDI1**, it is likely that the compounds inhibit NMD by the same mechanism, and therefore, that **VG1** also inhibits the interaction between UPF1 and SMG5, although this still needs to be confirmed. It is conceivable that **NMDI1** is converted into **VG1** in the cell and that the active form of the compounds is the same. The compound **VG1** passed the filter on an online PAINS (pan assay interference compounds) false positive remover tool,^39^ which helps to validate **VG1** as a genuine NMD inhibitor. Work is required to establish the mechanism of action of **VG1**. Whatever the outcome, **VG1** will be a useful tool for developing an understanding of both the mechanism of NMD and of other related mRNA decay pathways.
