# Supplementary material for: Synthesis and activity of a novel inhibitor of nonsense-mediated mRNA decay
Source: Org Biomol Chem. 2016 Jan 7;14(5):1559–63. doi: 10.1039/c5ob02482j (PMC4730866; doi:10.1039/c5ob02482j)
Supplement: Supplementary file 1 [file OB-014-c5ob02482j-s001.pdf]

Electronic Supplementary Material (ESI) for Organic & Biomolecular Chemistry.
This journal is © The Royal Society of Chemistry 2016

Supplementary Information

Synthesis and activity of a novel inhibitor of nonsense-mediated mRNA decay

Victoria J. B. Gotham,[a,b] Melanie C. Hobbs,[a] Ryan Burgin,[a] David Turton,[b] Carl Smythe*[b] and Iain Coldham*[a]

[a]*Department of Chemistry, University of Sheffield, Sheffield, S3 7HF, UK.*
E-mail: i.coldham@sheffield.ac.uk
[b]*Department of Biomedical Science, University of Sheffield, Sheffield, S10 2TN, UK.*

Contents Page

1. Chemistry – general experimental details S-2
2. First attempted route to NMDI1 S-3
   (Scheme, Experimental methods and NMR spectra)
3. Synthesis of VG1 S-11
   (Scheme, Experimental methods and NMR spectra)
4. Chlorination on nitrogen atom S-19
   (Scheme, Experimental methods and NMR spectra)
5. Cell biology S-22
   [Experimental methods (Cell culture, transfection and drug treatments; RNA analysis; Immunoblotting) and Data]
6. References S-26

1. Chemistry – general experimental details

All reagents were obtained from commercial suppliers and were used without further purification unless otherwise specified. Dry CH_2Cl_2, DMF and THF were obtained from the Grubbs dry solvent system (models SPS-400-6 and SPS-200-6). DBU and DMA were freshly distilled from and to molecular sieves. Thin layer chromatography was performed on Merck 60 F_{254} silica gel plates and visualised by UV irradiation at 254 nm or by staining with an alkaline $KMnO_4$ dip. Flash column chromatography was performed using Fluorochem Silicagel LC301 (40-63 micron mesh) or Fluorochem LC301SP Silicagel 60A. ^{1}H NMR spectra were recorded on a Bruker AC250 (250 MHz), a Bruker AC400 (400 MHz) or a Bruker DPX-400 (400 MHz) instrument. Chemical shifts are reported in ppm with respect to the residual solvent peaks, with multiplicities given as s = singlet, d = doublet, t = triplet, q = quartet, m = multiplet, br = broad. Coupling constants (*J* values) are quoted to the nearest 0.5 Hz and are corrected. ^{13}C NMR spectra were recorded on the above Bruker instruments at either 62.5 MHz or 100.6 MHz. Low and high resolution (accurate mass) mass spectra were recorded on a Micromass Autospec instrument for electron impact (EI) and on a Walters LCT instrument for electro-spray (ES). Infrared spectra were recorded on either a Perkin Elmer Paragon 1000 or a Perkin Elmer Spectrum 100 FT-IR spectrometer. Only selected peaks are reported and absorption maxima are given to the nearest 5 cm^{-1}. Melting points were recorded on a Gallenkamp hot stage or a Linkam HFS91 heating stage used in conjunction with a TC92 controller and were uncorrected. Volumes used in work-ups are approximate.

2. First attempted route to NMDI1

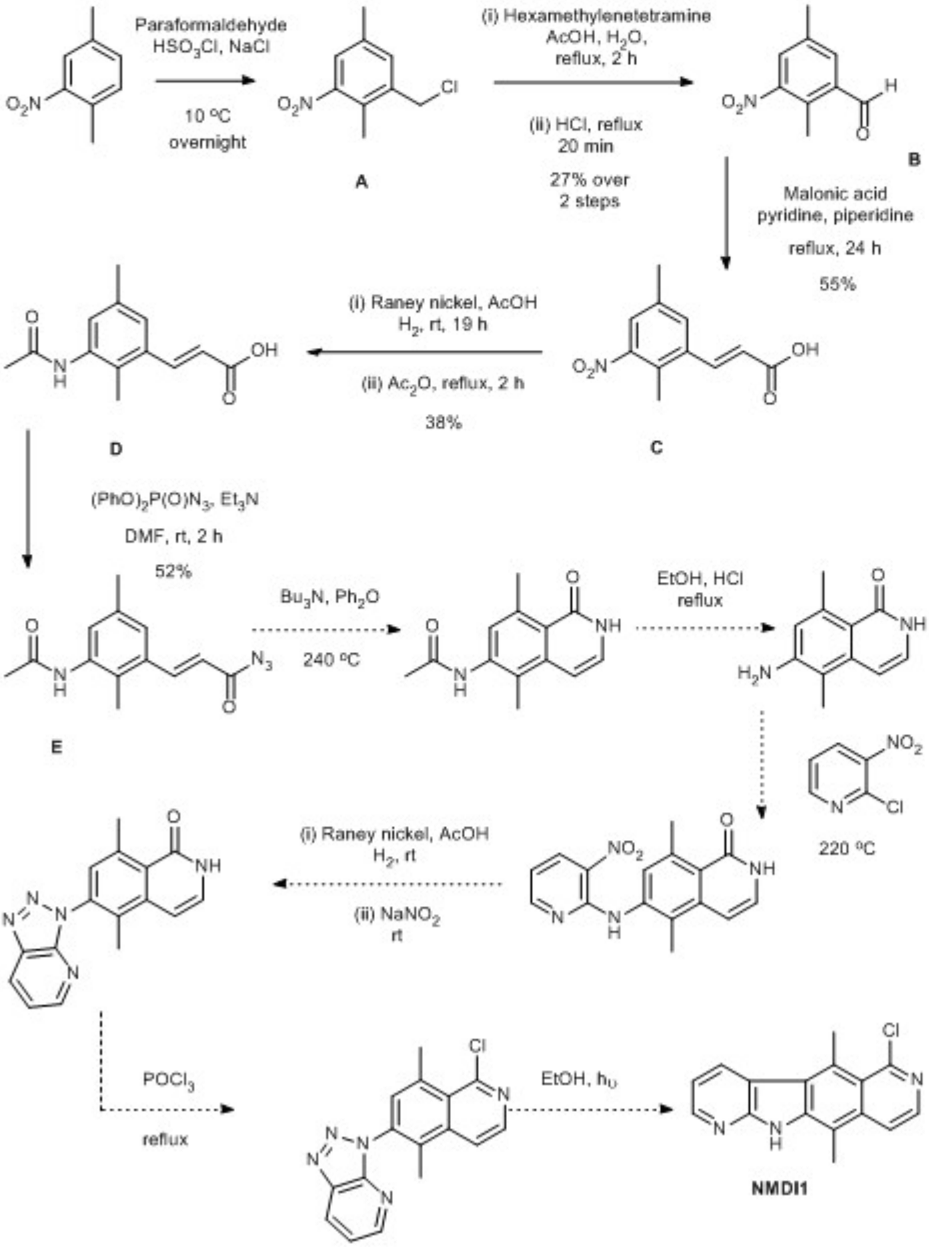

Scheme S1: First attempted synthesis of NMDI1, following the route used by Bisagni and co-workers.[1,2]

Experimental methods

1-Chloromethyl-2,5-dimethyl-3-nitrobenzene (A) and 1-chloromethyl-2,5-dimethyl-4-nitrobenzene

Paraformaldehyde (10 g, 330 mmol) was added to 2,5-dimethylnitrobenzene (8.85 mL, 66.2 mmol) and the mixture was stirred until a homogeneous paste was formed. Chlorosulfonic acid (23.0 mL, 346 mmol) was added between 10 and 20 °C dropwise over the course of a day. NaCl (4.0 g, 68 mmol) was added slowly at 10 °C and the reaction was allowed to warm to r.t. over 16 h. The mixture was cautiously added to ice-water (150 mL) and the crude product was extracted with $CHCl_3$ (3 x 250 mL). The combined organic layers were washed with aqueous $NaHCO_3$ (250 mL), dried (K_2CO_3) and concentrated under reduced pressure. The mixture was subjected to flash column chromatography, eluting with EtOAc-petrol (0:1 to 2:98). All fractions were collected which contained compound **A** and regioisomer 1-chloromethyl-

2,5-dimethyl-4-nitrobenzene, which could not be separated. The mixture of regioisomers was an amorphous solid, with **A** and 1-chloromethyl-2,5-dimethyl-4-nitrobenzene in a ratio of 14:1 (M:m): R_f [petrol] 0.30; ^{1}H NMR (400 MHz, $CDCl_3$) δ = 7.81 (1H, s, ArH^m), 7.54 (1H, s, ArH^M), 7.36 (1H, s, ArH^M), 7.29 (1H, s, ArH^m), 4.60 (2H, s, CH_2^M), 4.56 (2H, s, CH_2^m), 2.55 (3H, s, CH_3^m), 2.45 (3H, s, CH_3^M), 2.44 (3H, s, CH_3^m), 2.36 (3H, s, CH_3^M); ^{13}C NMR (100.6 MHz, $CDCl_3$) δ = 151.4 (C^M), 148.8 (C^m), 140.8 (C^m), 138.1 (C^M), 137.2 (C^M), 136.3 (C^m), 134.8 (CH^M), 134.0 (CH^m), 131.6 (C^m), 128.6 (C^M), 126.6 (CH^m), 124.9 (CH^M), 44.1 (CH_2^M), 43.1 (CH_2^m), 20.7 (CH_3^M), 20.0 (CH_3^m),18.3 (CH_3^m), 14.2 (CH_3^M); LRMS *m/z* (EI) 201 (3%, M$^+$, ^{37}Cl), 199 (8%, M$^+$, ^{35}Cl), 84 (100%); HRMS (EI) found: M$^+$ 199.0395, $C_9H_{10}NO_2{}^{35}Cl$ requires M$^+$ 199.0400. The mixture of regioisomers was taken on into the next step of the synthesis.

2,5-Dimethyl-3-nitrobenzaldehyde (B) and 2,5-dimethyl-4-nitro-benzaldehyde

AcOH (28 mL), water (28 mL) and hexamethylenetetramine (18.55 g, 132.3 mmol) were added to the mixture of regioisomers **A** and 1-chloromethyl-2,5-dimethyl-4-nitrobenzene and the mixture was heated under reflux for 2 h. The mixture was cooled to r.t., aqueous HCl (22 mL, 35%) was added slowly and the mixture was heated under reflux for 20 min. The mixture was cooled to r.t., water (130 mL) was added and the resulting precipitate was extracted with CH_2Cl_2 (3 x 300 mL). The combined organic layers were dried (Mg_2SO_4) and concentrated under reduced pressure. Purification by flash column chromatography, eluting with EtOAc-petrol (1:19), gave aldehyde **B** and regioisomer 2,5-dimethyl-4-nitro-benzaldehyde as an amorphous solid (4.2 g, 35% total yield over two steps, yield of **B** only 27% over 2 steps). Compounds **B** and 2,5-dimethyl-4-nitro-benzaldehyde could not be separated by column chromatography and were obtained in a ratio of 10:3 (M:m): R_f [petrol:EtOAc (95:5)] 0.26; ^{1}H NMR (400 MHz, $CDCl_3$) δ = 10.34 (1H, s, CH^{M}O), 10.31 (1H, s, CH^{m}O), 7.85 (1H, s, ArH^M), 7.80 (1H, s, ArH^m), 7.78 (1H, s, ArH^M), 7.77 (1H, s, ArH^m), 2.71 (3H, s, CH_3^M), 2.70 (3H, s, CH_3^m), 2.60 (3H, s, CH_3^m), 2.47 (3H, s, CH_3^M); ^{13}C NMR (100.6 MHz, $CDCl_3$, selected peaks for major regioisomer) δ = 190.8 (CH^{M}O), 151.9 (C^M), 137.6 (C^M), 135.8 (C^M), 135.5 (CH^M), 131.2 (C^M), 129.2 (CH^M), 20.7 (CH_3^M), 13.6 (CH_3^M); LRMS and HRMS could not be obtained.

2,5-Dimethyl-3-nitrocinnamic acid (C) and 2,5-dimethyl-4-nitrocinnamic acid

Malonic acid (4.024 g, 38.67 mmol), piperidine (7.7 mL) and dry pyridine (54 mL) were added to the mixture of aldehydes **B** and 2,5-dimethyl-4-nitro-benzaldehyde (6.928 g, 38.67 mmol) and the mixture was heated under reflux for 1 h. The mixture was cooled to r.t., a second equivalent of malonic acid (4.024 g, 38.67 mmol) was added and the mixture was heated under reflux for 1 h. The mixture was again cooled to r.t., a third equivalent of malonic acid (4.024 g, 38.67 mmol) was added and the mixture was heated under reflux for 15 h. The mixture was cooled to r.t. and concentrated under reduced pressure. CH_2Cl_2 (100 mL) was added and the carboxylate salt of the product was extracted with aqueous NaOH (3 x 100 mL, 2 M). The combined water layers were filtered and acidified to pH<1 by addition of aqueous HCl (35%). The resulting precipitate was filtered under vacuum, washed with aqueous HCl (100 mL, 2M), air-dried and dried under vacuum to give cinnamic acid **C** and regioisomer 2,5-dimethyl-4-nitrocinnamic acid as an amorphous solid (6.26 g, combined yield 73%, yield of **C** 55%). Compounds **C** and 2,5-dimethyl-4-nitrocinnamic acid were obtained in a ratio of 3:1 (M:m): R_f [petrol:EtOAc (85:15) with several drops of AcOH] 0.11; ^{1}H NMR (250 MHz, DMSO-d_6) δ = 12.68 (1H, s, OH^M), 12.68 (1H, s, OH^m), 7.90 (1H, s, ArH^m), 7.86 (1H, s, ArH^m), 7.83 (1H, s, ArH^M), 7.81 (1H, d, *J* 16 Hz, Ar-CH^M=), 7.74 (1H, d, *J* 16 Hz, Ar-CH^m=), 7.70 (1H, s, ArH^M), 6.61 (1H, d, *J* 16 Hz, =CH^mCO_2H), 6.51 (1H, d, *J* 16 Hz, =CH^MCO_2H), 2.50 (3H, s, CH_3^m), 2.42 (3H, s, CH_3^m), 2.36 (3H, s, CH_3^M), 2.35 (3H, s, CH_3^M); ^{13}C NMR (100.6 MHz, DMSO-d_6, selected peaks for major regioisomer) δ = 167.2 (C=O), 151.2 (C), 139.9 (CH), 137.2 (C), 135.7 (C), 131.4 (CH), 126.9 (C), 124.9 (CH), 123.4 (CH), 20.1 (CH_3), 14.2 (CH_3); LRMS *m/z* (ES) 266 (100%, MH$^+$); HRMS (ES) found: MH$^+$ 220.0606, $C_{11}H_{10}NO_2$ requires MH$^+$ 220.0610.

3-Acetamido-2,5-dimethylcinnamic acid (D)

AcOH (13 mL) was added to the mixture of cinnamic acids **C** and 2,5-dimethyl-4-nitrocinnamic acid (1.50 g, 6.78 mmol). Raney nickel (approximately 3 mL of slurry) was added and the mixture was stirred under

an H_2 atmosphere for 19 h. Use of an H_2 balloon without evacuating the flask to remove the air prior to adding the balloon avoided reduction of the double bond as well as the nitro group. The mixture was filtered and the catalyst was washed with AcOH (50 mL). The solution was concentrated to approximately 6 mL, Ac_2O (1.7 mL) was added, and the solution was heated under reflux for 2 h. The mixture was cooled to r.t. and concentrated under reduced pressure. Aqueous HCl (100 mL, 1 M) was added, the mixture was filtered and the solid was washed with aqueous HCl (20 mL, 1 M) and air-dried to give crude acid **D** and regioisomer 4-acetamido-2,5-dimethylcinnamic acid (1.14 g). Recrystallisation from AcOH gave single regioisomer **D** (0.451 g, 38% based on **C**) as microcrystals: mp 285 °C (lit.[1] mp 270 °C); R_f [petrol:EtOAc (1:1) with a few drops of AcOH] 0.26; ^{1}H NMR (400 MHz, DMSO-d_6) δ = 12.43 (1H, s, OH), 9.45 (1H, s, NH), 7.85 (1H, d, *J* 16 Hz, Ar-CH=), 7.38 (1H, s, ArH), 7.19 (1H, s, ArH), 6.39 (1H, d, *J* 16 Hz, =$CHCO_2H$), 2.28 (3H, s, CH_3), 2.18 (3H, s, CH_3), 2.06 (3H, s, CH_3); ^{13}C NMR (100.6 MHz, DMSO-d_6) δ = 168.3 (C=O), 167.5 (C=O), 141.7 (CH), 136.9 (C), 135.0 (C), 133.7 (C), 129.0 (C), 128.2 (CH), 124.4 (CH), 120.6 (CH), 23.1 (CH_3), 20.4 (CH_3), 13.6 (CH_3); LRMS *m/z* (ES) 234 (100%, MH^+); HRMS (ES) found: MH^+ 234.1124, $C_{13}H_{16}NO_3$ requires MH^+ 234.1130.

3-Acetamido-2,5-dimethylcinnamoyl azide (E)

Cinnamic acid **D** (0.10 g, 0.43 mmol) was suspended in dry DMF (0.3 mL) and was dissolved on addition of Et_3N (0.1 mL, 0.7 mmol). $(PhO)_2PON_3$ (0.093 mL, 0.43 mmol) and additional dry DMF (0.3 mL) were added and the mixture was stirred at r.t. for 2 h. Water (1.5 mL) was added and the resulting precipitate was filtered, washed with water (1 mL) and acetone (1 mL) and thoroughly air-dried to give acyl azide **E** (57 mg, 52 %) as an amorphous solid: R_f [petrol:EtOAc (7:3)] 0.12; IR $υ_{max}$ (solid)/cm^{-1} 3270, 2145, 1680, 1655, 1530, 1455, 1245, 1200; ^{1}H NMR (400 MHz, DMSO-d_6) δ = 9.47 (1H, s, NH), 7.99 (1H, d, *J* 16 Hz, Ar-CH=), 7.47 (1H, s, ArH), 7.22 (1H, s, ArH), 6.56 (1H, d, *J* 16.0 Hz, =$CHCON_3$), 2.27 (3H, s, CH_3), 2.19 (3H, s, CH_3), 2.04 (3H, s, CH_3); ^{13}C NMR (100.6 MHz, DMSO-d_6) δ = 171.5 (C=O), 168.3 (C=O), 144.2 (CH), 137.1 (C), 135.2 (C), 132.9 (C), 129.9 (C), 129.2 (CH), 124.8 (CH), 120.3 (CH), 23.1 (CH_3), 20.4 (CH_3), 13.5 (CH_3); LRMS *m/z* (ES) 259 (100%, MH^+), 231 (60%); HRMS (ES) found: MH^+ 259.1197, $C_{13}H_{15}N_4O_2$ requires MH^+ 259.1195.

Attempted cyclisation of acyl azide **E** by the method used by Bisagni and co-workers[1] failed to give the desired lactam product.

NMR spectra

1-Chloromethyl-2,5-dimethyl-3-nitrobenzene (**A**) and 1-chloromethyl-2,5-dimethyl-4-nitrobenzene

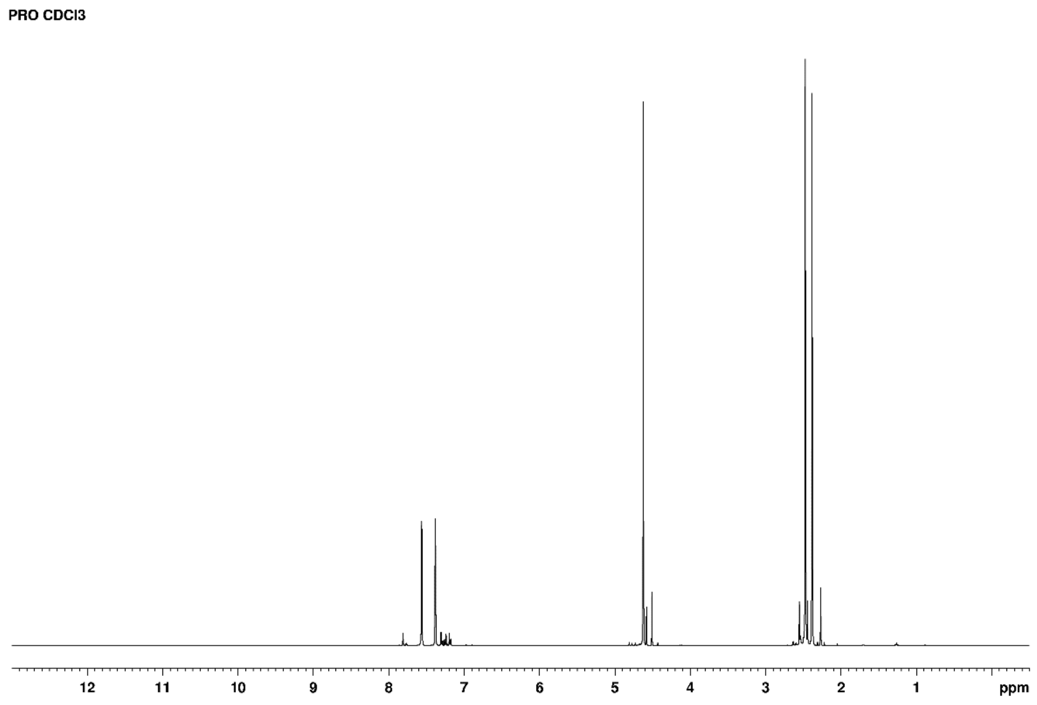

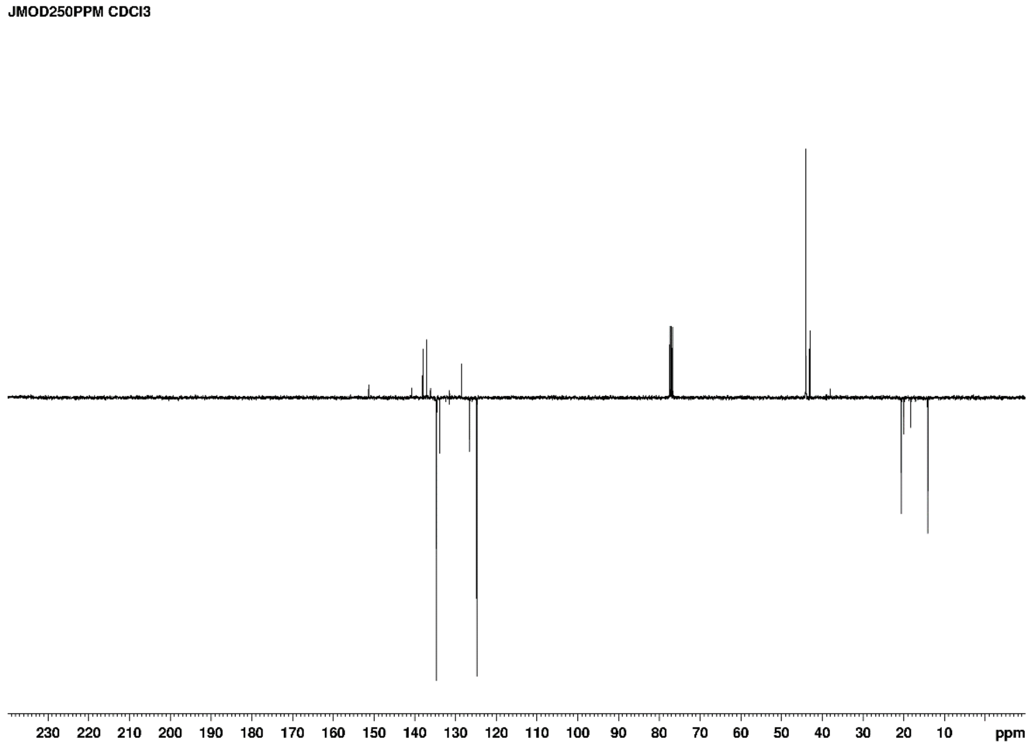

2,5-Dimethyl-3-nitrobenzaldehyde (**B**) and 2,5-dimethyl-4-nitro-benzaldehyde

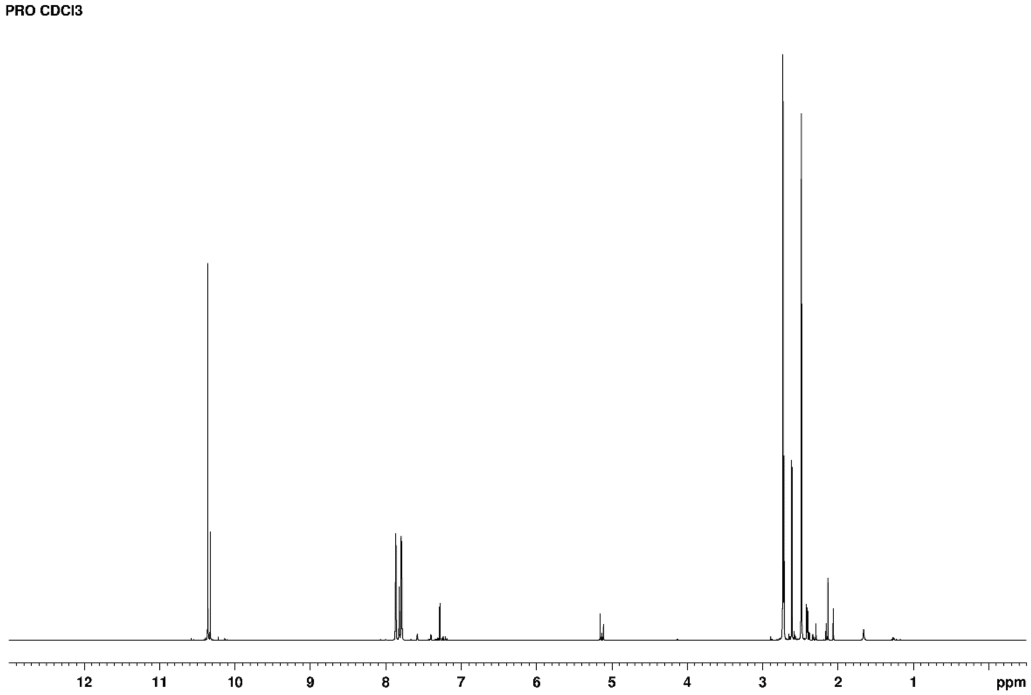

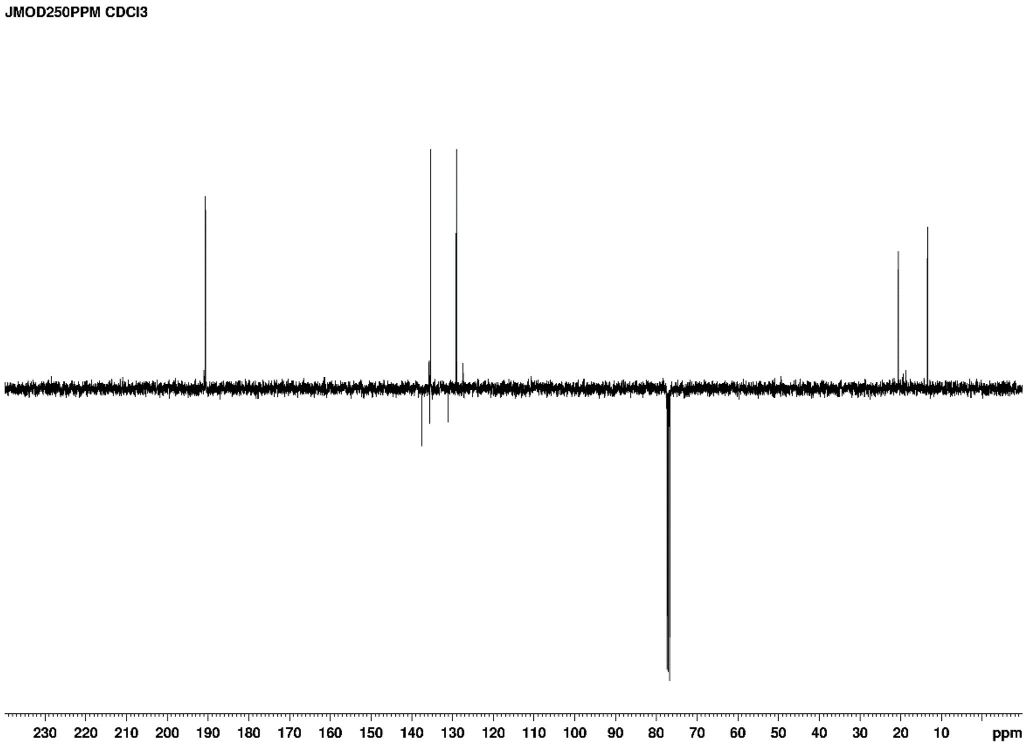

2,5-Dimethyl-3-nitrocinnamic acid (C) and 2,5-dimethyl-4-nitrocinnamic acid

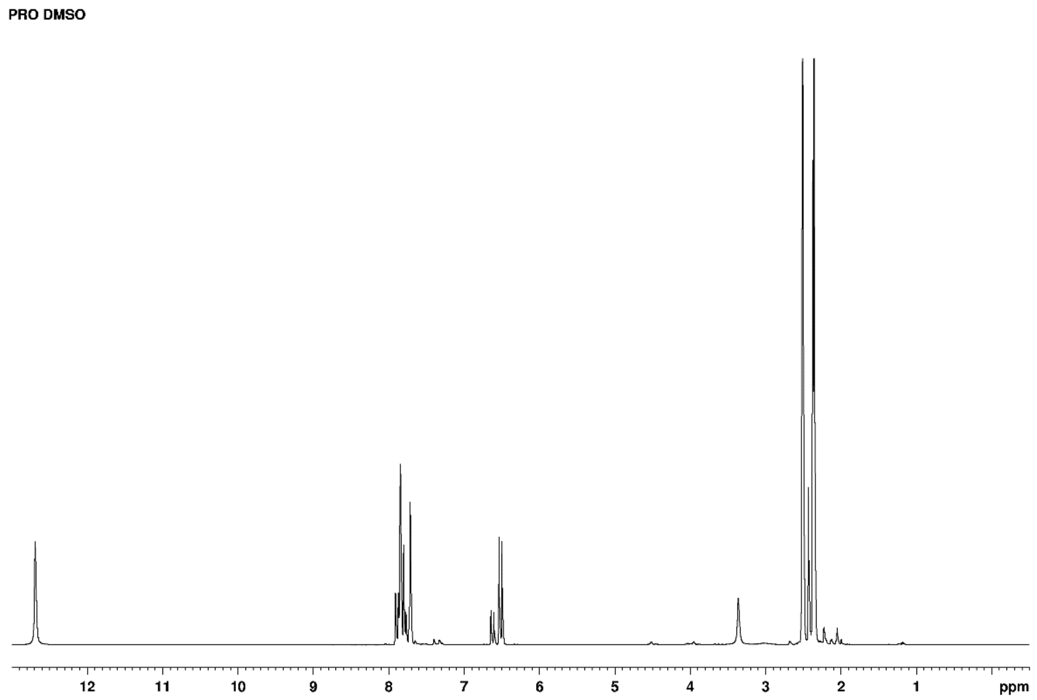

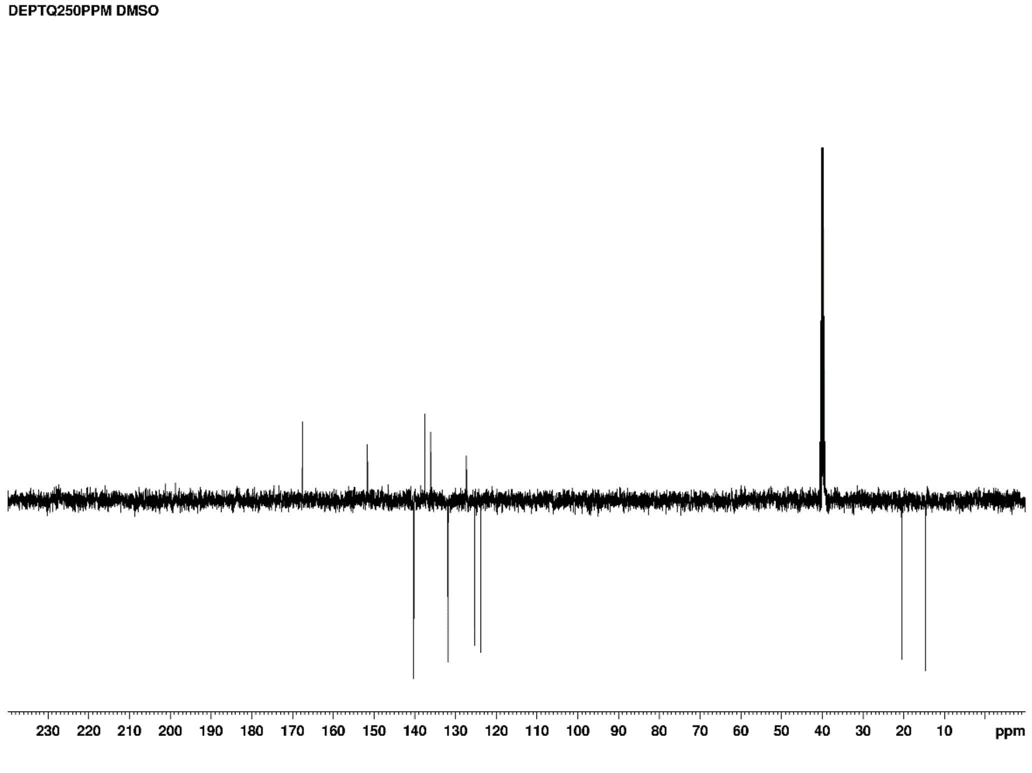

3-Acetamido-2,5-dimethylcinnamic acid (**D**)

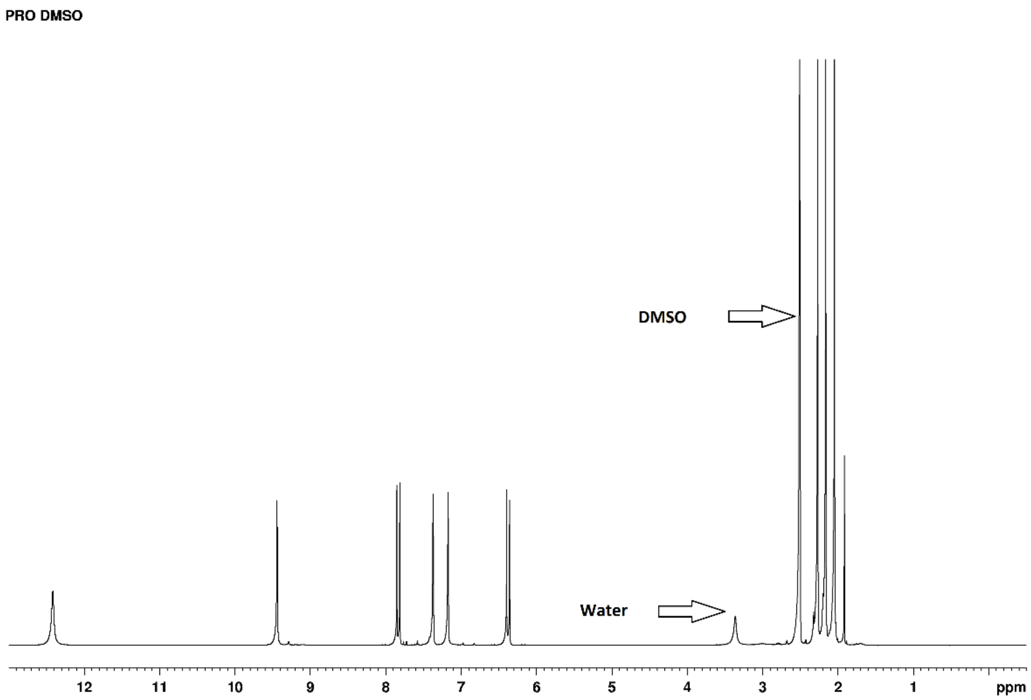

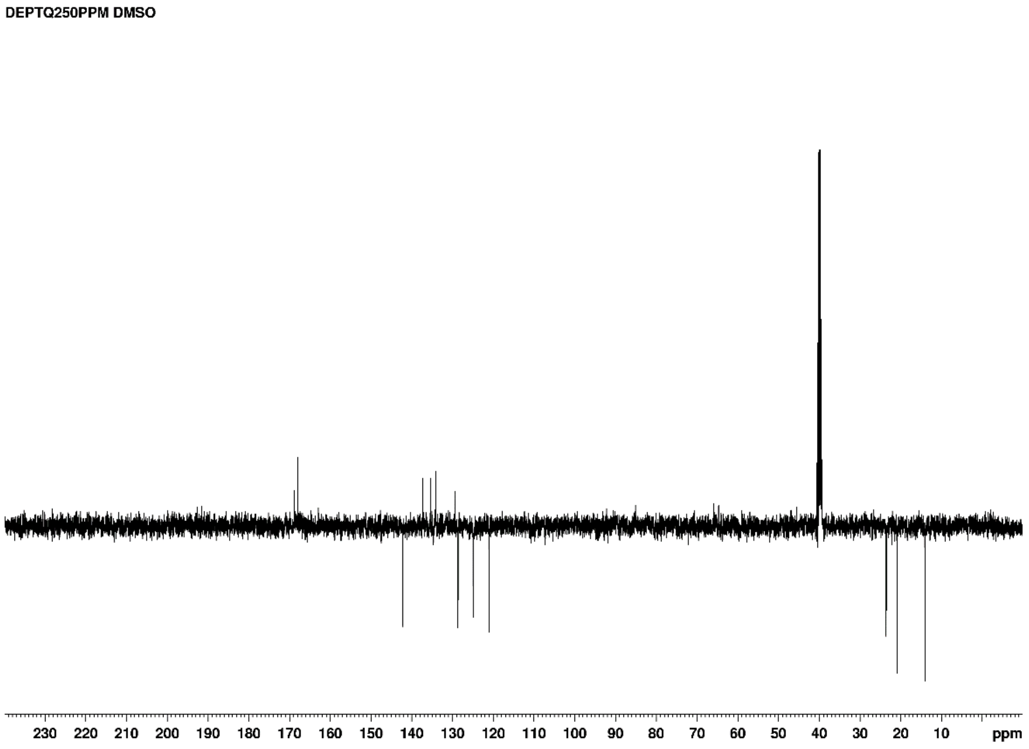

3-Acetamido-2,5-dimethylcinnamoyl azide (**E**)

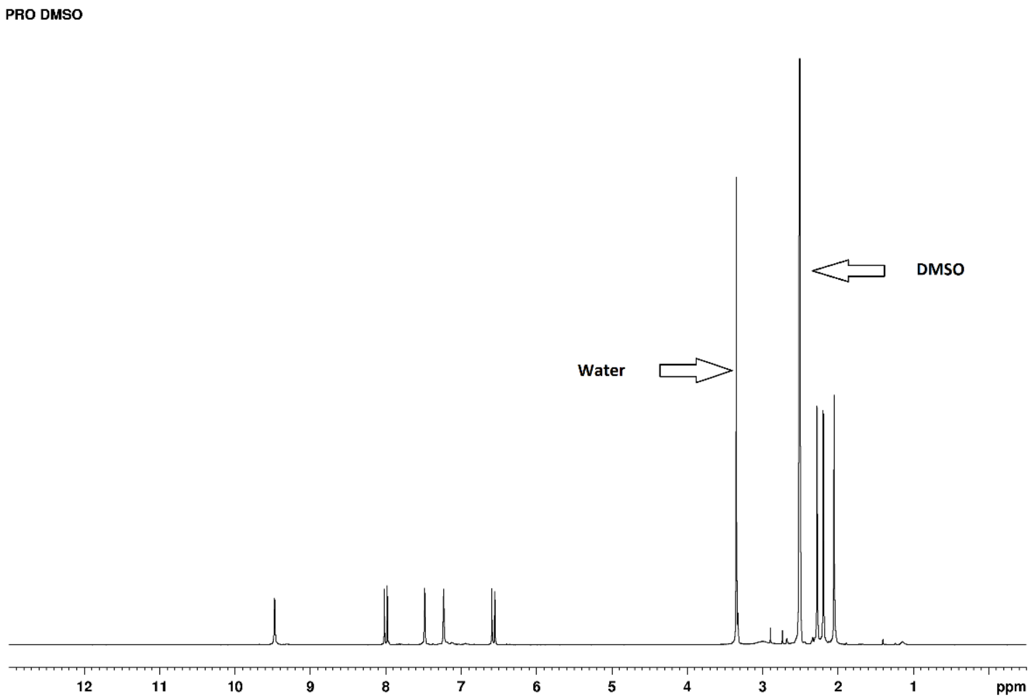

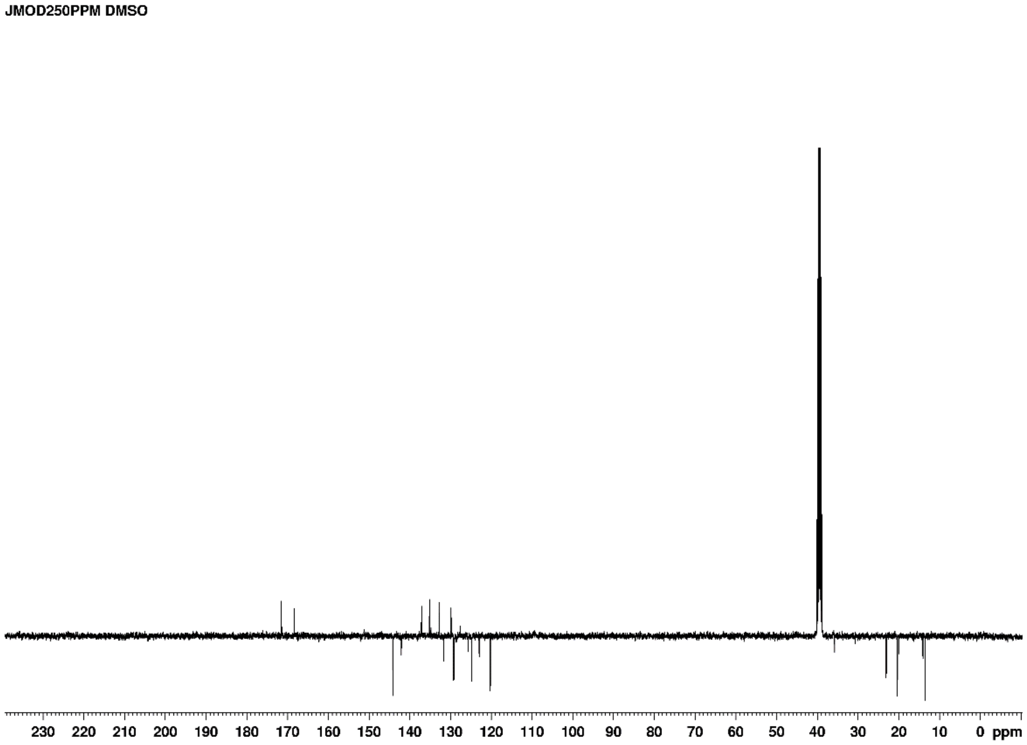

3. Synthesis of VG1

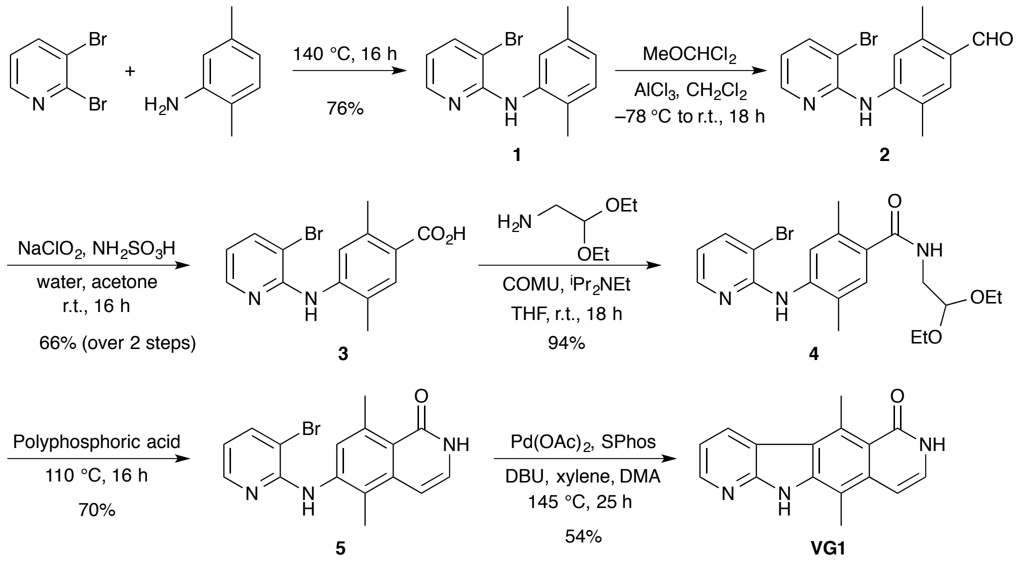

Scheme S2: Synthesis of VG1.

Experimental methods

N-(2,5-Dimethylphenyl)-2-amino-3-bromopyridine (1)

2,3-Dibromopyridine (23.5 g, 99 mmol) and 2,5-dimethylaniline (12.4 mL, 99 mmol) were heated at 140 °C for 16 h. CH_2Cl_2 (200 mL) and NaOH (200 mL, 2 M) were added, the layers were separated and the crude product was extracted with CH_2Cl_2 (3 x 100 mL). The combined organic layers were dried (Mg_2SO_4) and concentrated under reduced pressure. Purification by flash column chromatography, eluting with EtOAc-petrol (2:98), gave amine **1** (20.9 g, 76%) as an amorphous solid: mp 41–45 °C; R_f [petrol:EtOAc (98:2)] 0.2; IR υ_{max} (solid)/cm^{-1} 3425, 3040, 3005, 2910, 2855, 1590, 1520, 1450, 1425, 1400, 1110, 1010; ^{1}H NMR (400 MHz, $CDCl_3$) δ = 8.22 (1H, dd, *J* 5, 1.5 Hz, ArH), 7.90 (1H, s, ArH), 7.76 (1H, dd, *J* 7.5, 1.5 Hz), 7.20 (1H, d, *J* 7.5 Hz, ArH), 6.96 (1H, d, *J* 7.5 Hz, ArH), 6.88 (1H, s, NH), 6.64 (1H, dd, *J* 7.5, 5 Hz, ArH), 2.46 (3H, s, CH_3), 2.36 (3H, s, CH_3); ^{13}C NMR (100.6 MHz, $CDCl_3$) δ = 152.4 (C), 146.6 (CH), 140.0 (CH), 137.7 (C), 136.2 (C), 130.3 (CH), 126.3 (C), 124.6 (CH), 122.6 (CH), 115.0 (CH), 106.4 (C), 21.3 (CH_3), 17.6 (CH_3); LRMS *m/z* (ES) 279 (100%, MH^+, ^{81}Br), 277 (100%, MH^+, ^{79}Br); HRMS (ES) found: MH^+ 277.0354, $C_{13}H_{14}N_2{}^{79}Br$ requires MH^+ 277.0340.

4-(3-Bromopyridin-2-ylamino)-2,5-dimethylbenzoic acid (3)

Bromopyridine **1** (4 g, 14.7 mmol) was dissolved in dry CH_2Cl_2 (26 mL) and cooled to -78 °C. $AlCl_3$ (13.7 g, 102.9 mmol) was added portionwise and the mixture was stirred for 5 min. $MeOCHCl_2$ (4 mL, 44.1 mmol) was added dropwise. The mixture was stirred for 18 h whilst being allowed to warm to r.t.. The mixture was poured cautiously into ice-water (80 mL) and the crude aldehyde **2** was extracted with CH_2Cl_2 (3 x 200 mL). The combined organic layers were dried (Mg_2SO_4), filtered and concentrated under reduced pressure. The mixture was suspended in a mixture of acetone (80 mL) and water (290 mL). NH_2SO_3H (2.07 g, 21.3 mmol) and $NaClO_2$ (2.13 g, 23.5 mmol) were added and the mixture was stirred for 16 h at r.t.. The crude product was extracted with EtOAc (3 x 200 mL) and the carboxylate salt of the product was extracted from EtOAc with aqueous NaOH (4 x 300 mL, 2 M). The combined NaOH layers were acidified to approximately pH 5 using conc. HCl and the resulting precipitate was filtered, air-dried, and dried under vacuum to give acid **3** (3.14 g, 66%) as an amorphous solid; mp 195–199 °C; R_f [petrol:EtOAc (95:5) with a several drops of AcOH] 0.3; IR υ_{max} (solid)/cm^{-1} 3414, 2970–2540, 1665, 1585, 1520, 1450, 1400, 1340,

1245, 1140, 1005; ^{1}H NMR (400 MHz, $CDCl_3$) δ = 10.86 (1H, br s, COOH), 8.32 (1H, s, ArH), 8.24 (1H, dd, *J* 5, 1.5 Hz, ArH), 7.95 (1H, s, ArH), 7.80 (1H, dd, *J* 8.0, 1.5 Hz, ArH), 7.14 (1H, s, NH), 6.73 (1H, dd, *J* 8.0, 5 Hz, ArH), 2.67 (3H, s, CH_3), 2.37 (3H, s, CH_3); ^{13}C NMR (100.6 MHz, DMSO-d_6) δ = 172.4 (C=O), 169.5 (C), 152.4 (C), 146.5 (CH), 140.8 (CH), 140.6 (C), 136.6 (C), 132.2 (CH), 127.5 (C), 125.5 (CH), 115.9 (CH), 106.1 (C), 21.3 (CH_3), 17.4 (CH_3); LRMS *m/z* (ES) 323 (100%, MH^+, ^{81}Br), 321 (95%, MH^+, ^{79}Br); HRMS (ES) found: MH^+ 321.0232, $C_{14}H_{14}N_2O_2Br$ requires MH^+ 321.0239.

4-(3-Bromopyridin-2-ylamino)-*N*-(2,2-diethoxyethyl)-2,5-dimethylbenzamide (4)

Acid **3** (7.46 g, 23 mmol) was dissolved in dry THF (240 mL). $^iPr_2NEt$ (5.0 mL, 29 mmol) and COMU (10.4 g, 24.2 mmol) were added and the mixture was stirred at r.t. for 2 h. $NH_2CH_2CH(OEt)_2$ (3.4 mL, 23 mmol) was added and the mixture was stirred at r.t. for 18 h. The mixture was concentrated under reduced pressure and water was added (100 mL). The crude product was extracted with Et_2O (3 x 100 mL) and the combined organic layers were dried (Mg_2SO_4), filtered and concentrated under reduced pressure. Purification by flash column chromatography, eluting with AcOH-EtOAc-petrol (1:59:40), gave amide **4** (9.41 g, 94%) as an amorphous solid; mp 99-102 °C; R_f [petrol:EtOAc (3:2)] 0.24; IR $υ_{max}$ (solid)/cm^{-1} 3420, 3275, 2975, 2925, 1635, 1600, 1515, 1450, 1410, 1345, 1120, 1065, 1005; ^{1}H NMR (400 MHz, $CDCl_3$) δ = 8.16 (1H, dd, *J* 5.0, 1.5 Hz, ArH), 7.96 (1H, s, ArH), 7.75 (1H, dd, *J* 8.0, 1.5 Hz, ArH), 7.28 (1H, s, ArH), 6.89 (1H, s, NH), 6.65 (1H, dd, *J* 8.0, 5.0 Hz, ArH), 5.99 (1H, t, *J* 5.5 Hz, NH), 4.62 (1H, t, *J* 5.5 Hz, CH), 3.79-3.70 (2H, m, CH_2), 3.63-3.54 (4H, m, 2 x CH_2), 2.47 (3H, s, CH_3), 2.29 (3H, s, CH_3), 1.24 (6H, t, *J* 7.0 Hz, 2 x CH_3); ^{13}C NMR (100.6 MHz, $CDCl_3$) δ = 169.9 (C=O), 151.9 (C), 146.7 (CH), 140.3 (CH), 139.5 (C), 134.9 (C), 130.9 (C), 129.5 (CH), 125.6 (C), 123.1 (CH), 115.9 (CH), 106.9 (C), 100.9 (CH), 62.9 (CH_2), 42.3 (CH_2), 20.2 (CH_3), 17.6 (CH_3), 15.4 (CH_3); LRMS *m/z* (ES) 438 (80%, MH^+, ^{81}Br), 436 (100%, MH^+, ^{79}Br); HRMS (ES) found: MH^+ 436.1227, $C_{20}H_{27}N_3O_3Br$ requires MH^+ 436.1236.

6-(3-Bromopyridin-2-ylamino)-5,8-dimethylisoquinolin-1(2*H*)-one (5)

Polyphosphoric acid (80 g) was heated to 110 °C. Amide **4** (2.00 g, 4.58 mmol) was added portionwise and the mixture stirred for 16 h at 110 °C. The mixture was poured onto ice-water (250 mL) and the crude product was extracted with $CHCl_3$ (3 x 250 mL). The combined organic layers were dried (Na_2SO_4), filtered and concentrated under reduced pressure. Purification by flash column chromatography, eluting with EtOAc-petrol (10:90), gave lactam **5** (1.10 g, 70%) as an amorphous solid; mp 89–91 °C; R_f [petrol:EtOAc (9:1)] 0.21; IR $υ_{max}$ (solid)/cm^{-1} 3425, 3135, 2915, 2855, 1700, 1595, 1555, 1520, 1480, 1450, 1410, 1360, 1250, 1120, 1105, 1005; ^{1}H NMR (400 MHz, $CDCl_3$) δ = 8.22-8.18 (1H, m, ArH), 8.19 (1H, s, NH), 7.86 (1H, s, ArH), 7.76 (1H, dd, *J* 8.0, 1.5 Hz, ArH), 7.70 (1H, d, *J* 0.5 Hz, CH), 7.24 (1H, d, *J* 0.5 Hz, CH), 7.00 (1H, br s, NH), 6.69 (1H, dd, *J* 8.0, 5.0 Hz, ArH), 2.70 (3H, s, CH_3), 2.36 (3H, s, CH_3); ^{13}C NMR (100.6 MHz, $CDCl_3$) δ = 162.8 (C=O), 151.8 (C), 146.7 (CH), 140.4 (CH), 139.8 (C), 137.8 (C), 136.3 (C), 131.0 (CH), 128.1 (CH), 125.0 (C), 122.5 (CH), 121.0 (C), 116.1 (CH), 107.2 (C), 22.1 (CH_3), 17.6 (CH_3); LRMS *m/z* (ES) 346 (95%, MH^+, ^{81}Br), 344 (100%, MH^+, ^{79}Br); HRMS (ES) found: MH^+ 344.0407, $C_{16}H_{15}N_3OBr$ requires MH^+ 344.0398.

6,11-Dimethyl-5*H*-pyrido[3',2':4,5]pyrrolo[2,3-g]isoquinolin-10(9*H*)-one (VG1)

Lactam **5** (0.20 g, 0.58 mmol), $Pd(OAc)_2$ (6.5 mg, 0.029 mmol), SPhos (23.8 mg, 0.058 mmol) and freshly-distilled DBU (0.18 mL, 1.2 mmol) were stirred in a mixture of dry *o*-xylene (1.5 mL) and freshly-distilled DMA (1.5 mL), degassed for 10 minutes with nitrogen and heated at 145 °C for 25 h. The mixture was cooled to r.t. and concentrated under reduced pressure. Purification by flash column chromatography, eluting with EtOAc-petrol (30:70), gave carboline **VG1** (82 mg, 54%) as an amorphous solid. A portion of the compound was further purified using preparative HPLC (35% MeCN in H_2O, 17 mL per min); mp 294–299 °C; R_f [petrol:EtOAc (3:2)] 0.23; IR $υ_{max}$ (solid)/cm^{-1} 3135, 3065, 2960, 2920, 2845, 1725, 1580, 1545, 1415, 1350, 1265, 1105, 1105, 1045; ^{1}H NMR (400 MHz, DMSO-d_6) δ = 12.14 (1H, s, NH), 8.60 (1H, dd, *J* 8.0, 1.0 Hz, ArH), 8.48 (1H, dd, *J* 5.0, 1.0 Hz, ArH), 8.22 (1H, d, *J* 0.5 Hz, CH), 7.79 (1H, s, NH), 7.40 (1H, d, *J* 0.5 Hz, CH), 7.27 (1H, dd, *J* 8.0, 5.0 Hz, ArH), 3.11 (3H, s, CH_3), 2.59 (3H, s, CH_3); ^{13}C NMR (100.6 MHz,

DMSO-d_6) δ = 162.0 (C=O), 152.3 (C), 145.8 (CH), 139.0 (C), 138.9 (C), 130.8 (C), 130.6 (CH), 127.9 (CH), 127.5 (CH), 119.6 (C), 118.6 (C), 118.0 (C), 116.2 (C), 115.4 (CH), 17.2 (CH_3), 16.6 (CH_3); LRMS *m/z* (ES) 264 (100%, MH^+); HRMS (ES) found: MH^+ 264.1140, $C_{16}H_{14}N_3O$ requires MH^+ 264.1137.

NMR spectra

N-(2,5-Dimethylphenyl)-2-amino-3-bromopyridine (**1**)

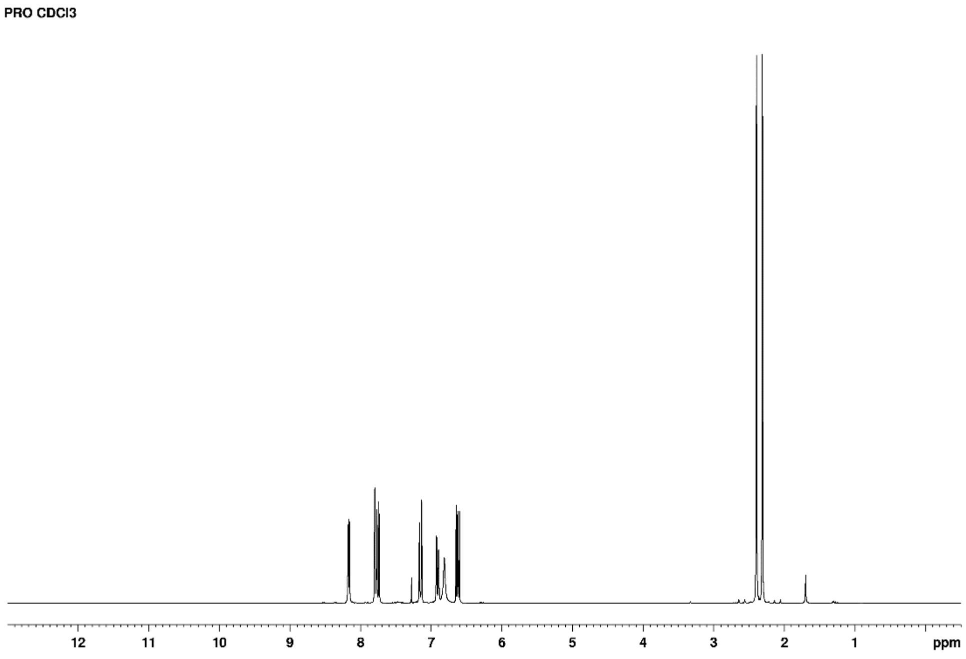

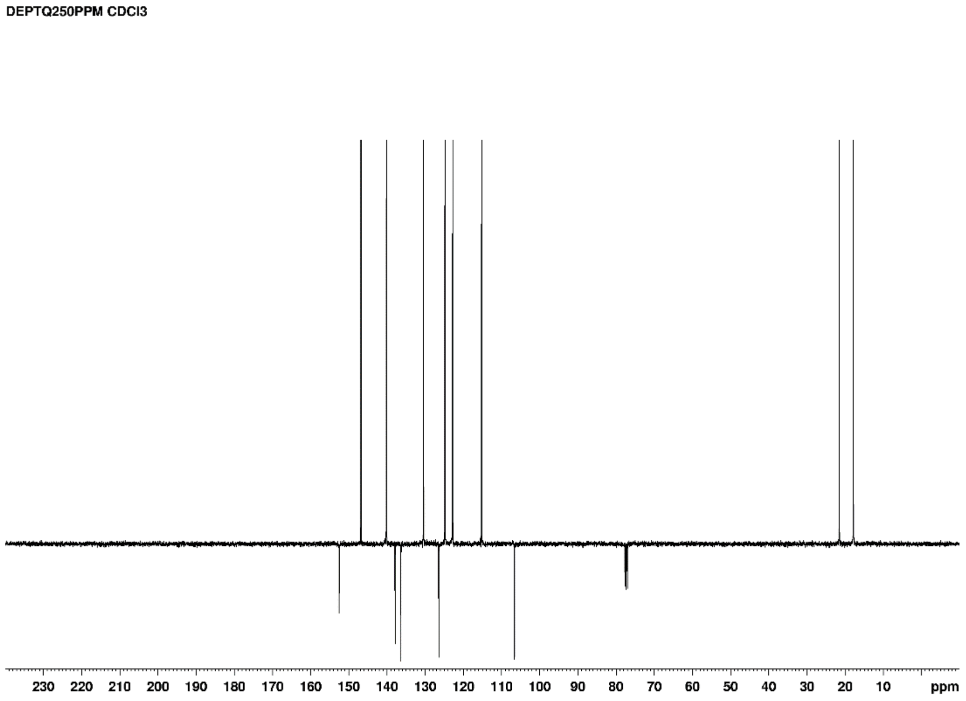

4-(3-Bromopyridin-2-ylamino)-2,5-dimethylbenzoic acid (**3**)

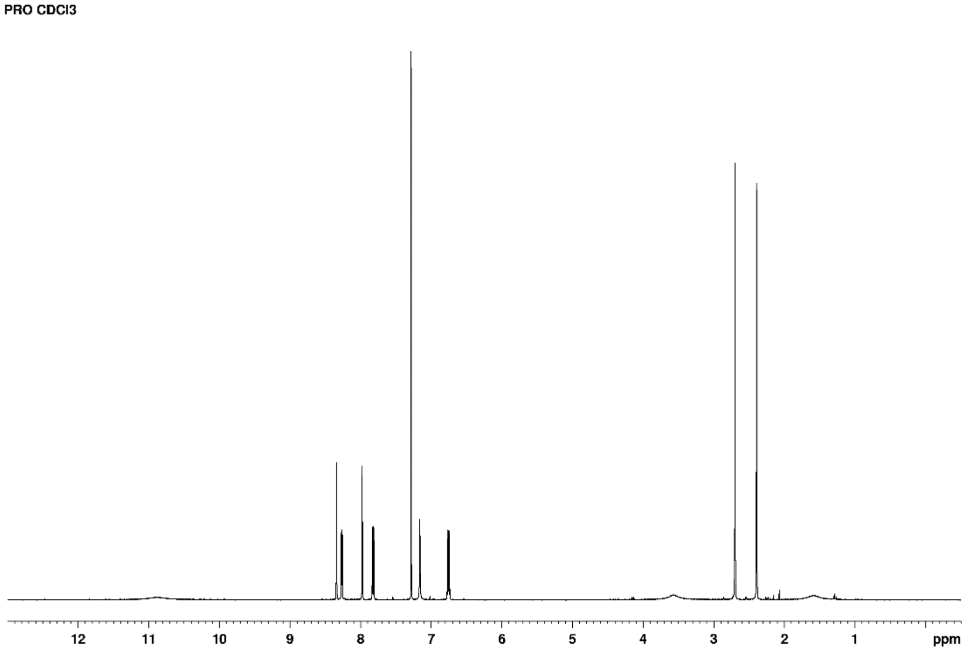

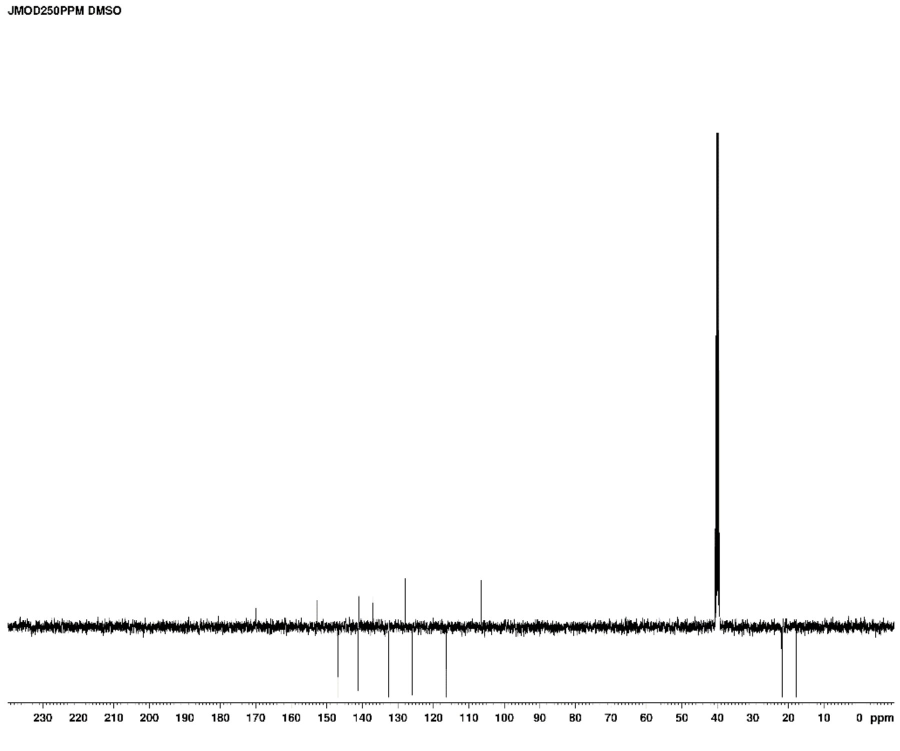

C13CPD250PPM DMSO

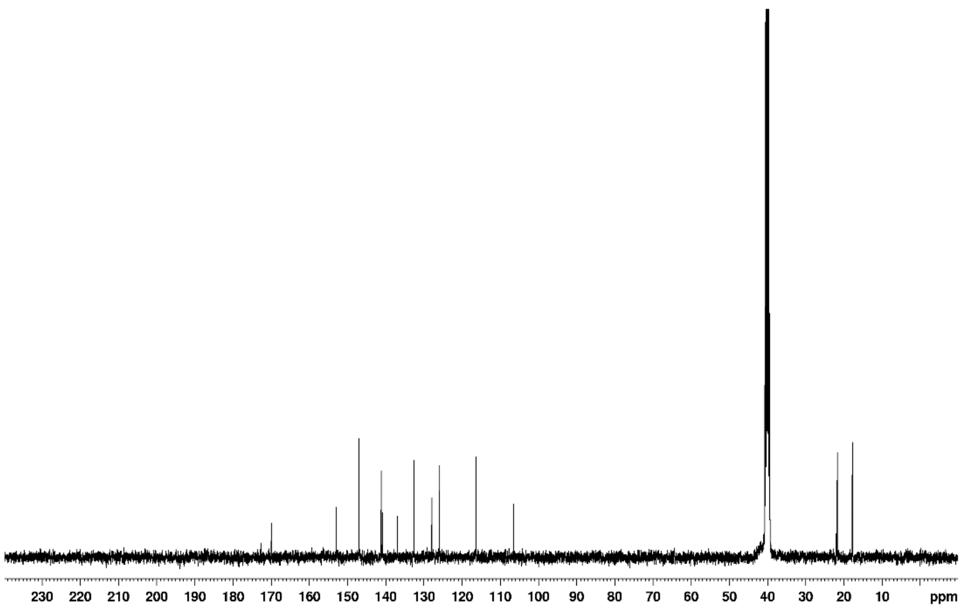

4-(3-Bromopyridin-2-ylamino)-*N*-(2,2-diethoxyethyl)-2,5-dimethylbenzamide (**4**)

PRO CDCl3

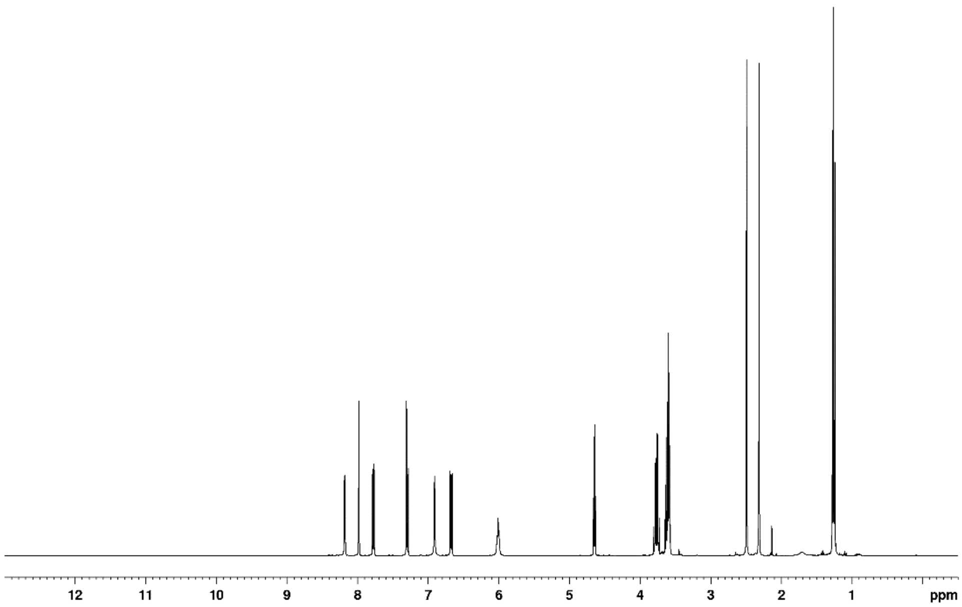

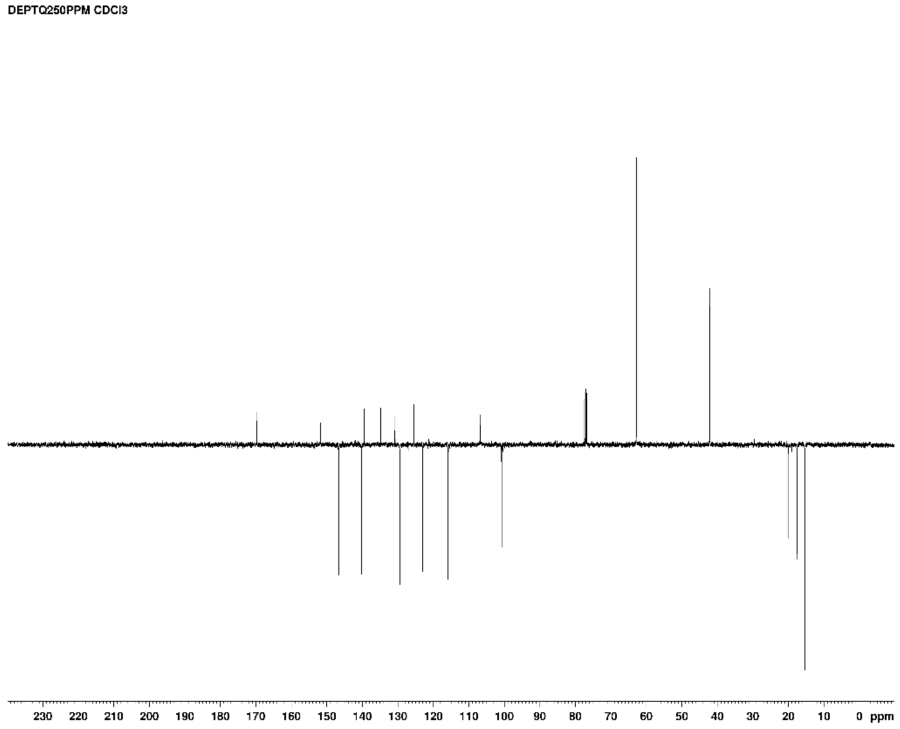

6-(3-Bromopyridin-2-ylamino)-5,8-dimethylisoquinolin-1(2*H*)-one (**5**)

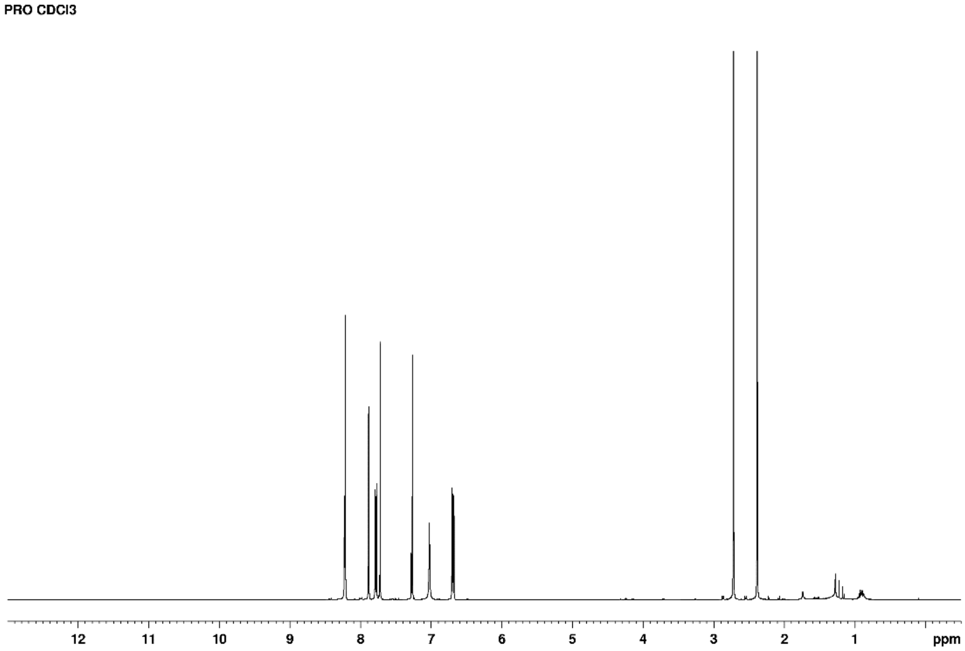

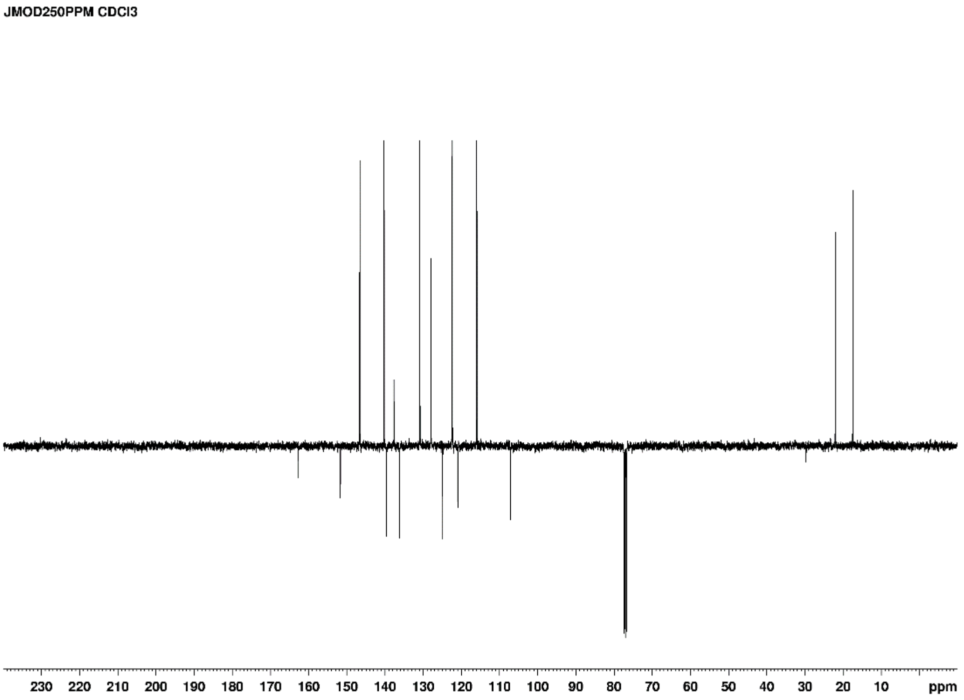

6,11-Dimethyl-5*H*-pyrido[3',2':4,5]pyrrolo[2,3-g]isoquinolin-10(9*H*)-one (**VG1**)

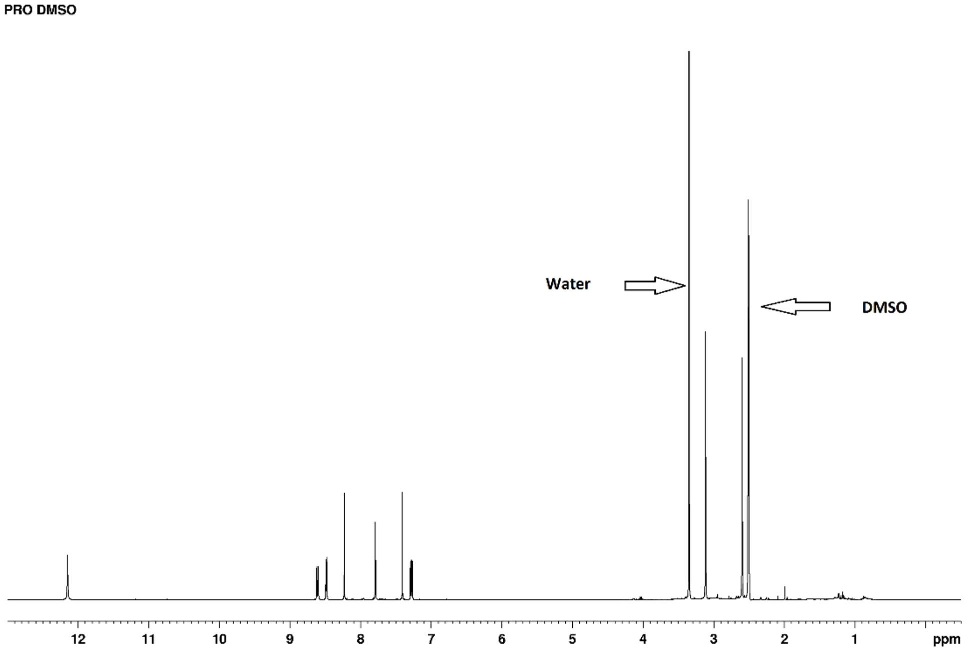

JMOD250PPM DMSO

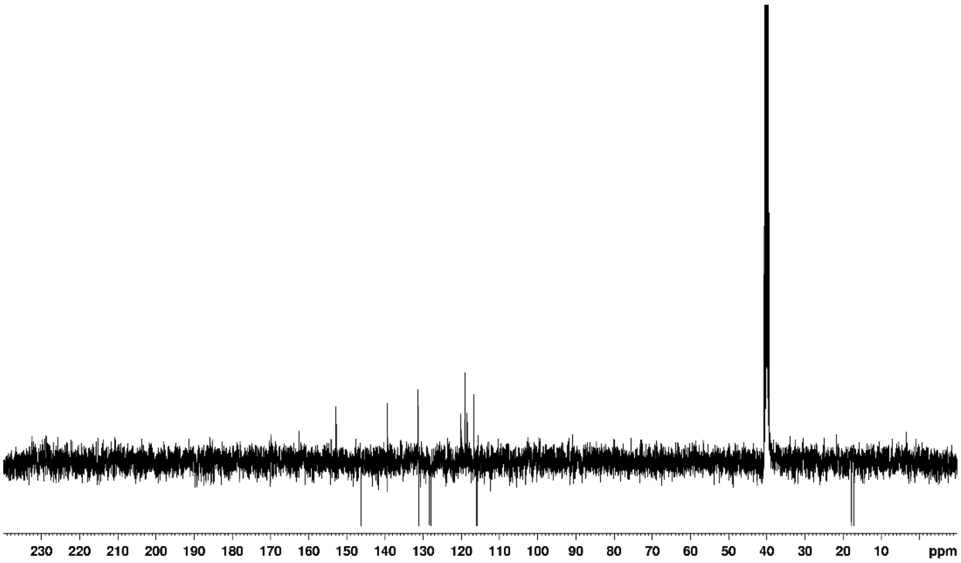

C13CPD250PPM DMSO

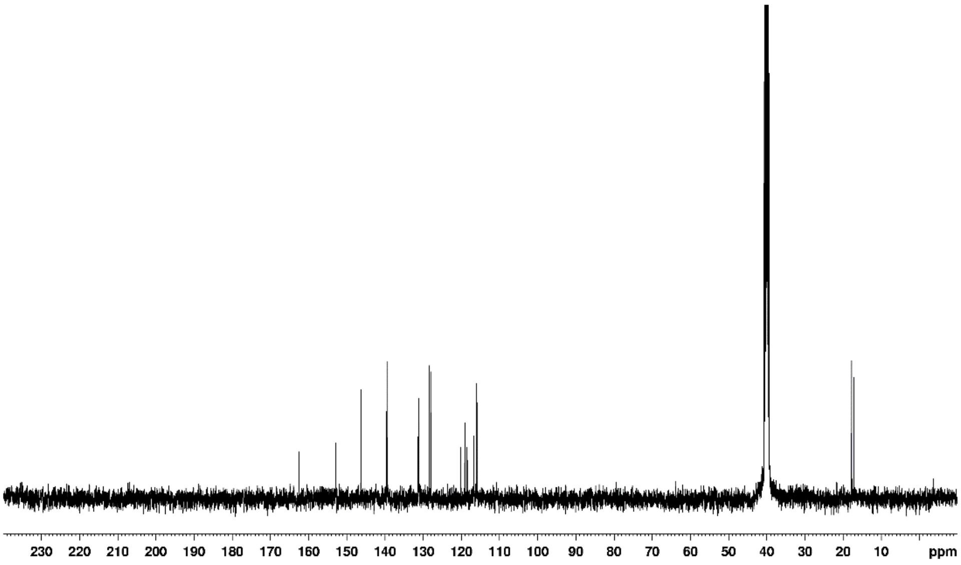

4. Chlorination on nitrogen atom

Treatment of compound 5 with PCl_5 in $POCl_3$ gave *N*-chloro derivative **F** (Scheme S3). Chlorination of **VG1** under the same conditions gave a low yield of impure *N*-chlorinated product.

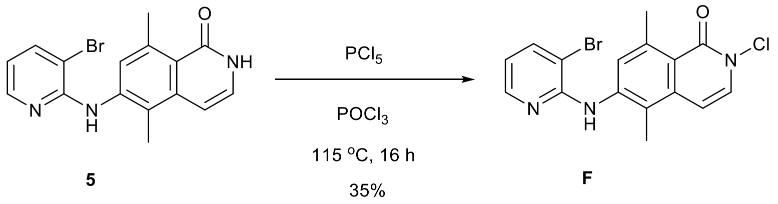

Scheme S3: Chlorination on nitrogen atom.

Experimental method

6-[(3-Bromopyridin-2-yl)amino]-2-chloro-5,8-dimethyl-1,2-dihydroisoquinolin-1-one (F)

Lactam **5** (50 mg, 0.15 mmol) and PCl_5 (0.03 mL, 0.145 mmol) in $POCl_3$ (8.5 mL) were heated at 115 °C overnight. The mixture was cooled to r.t., poured cautiously into ice-water (50 mL) and neutralised with solid K_2CO_3. The crude product was extracted with EtOAc (3 x 50 mL), dried (Mg_2SO_4), filtered and concentrated under reduced pressure. Recrystallisation from EtOH gave chloride **F** (19 mg, 35%) as microcrystals; mp 128–130 °C; R_f [petrol:EtOAc (9:1)] 0.3; IR υ_{max} (solid)/cm^{-1} 1595, 1520, 1450, 1410, 1105, 1005; ^{1}H NMR (400 MHz, $CDCl_3$) δ = 8.23 (1H, s, ArH or CH), 8.21 (1H, dd, *J* 5.0, 1.5 Hz, ArH), 7.80 (1H, s, ArH or CH), 7.78 (1H, dd, *J* 8.0, 1.5 Hz, ArH), 7.03 (1H, br s, NH), 7.00 (1H, s, ArH or CH), 6.69 (1H, dd, *J* 8.0, 5.0 Hz, ArH), 2.67 (3H, s, CH_3), 2.37 (3H, s, CH_3); ^{13}C NMR (100.6 MHz, $CDCl_3$) δ = 161.8 (C=O), 151.7 (C), 146.7 (CH), 140.5 (CH), 140.1 (C), 136.4 (C), 135.2 (C), 130.6 (CH), 124.9 (C), 123.5 (CH), 122.3 (CH), 120.0 (C), 116.3 (CH), 107.4 (C), 22.1 (CH_3), 17.6 (CH_3); LRMS *m/z* (ES) 382 (25%, MH^+, ^{81}Br, ^{37}Cl), 380 (100%, MH^+, ^{81}Br, ^{35}Cl and ^{79}Br, ^{37}Cl), 378 (75%, MH^+, ^{79}Br, ^{35}Cl); HRMS (ES) found: MH^+ 378.0006, $C_{16}H_{14}N_3OClBr$ requires MH^+ 378.0009.

NMR spectra

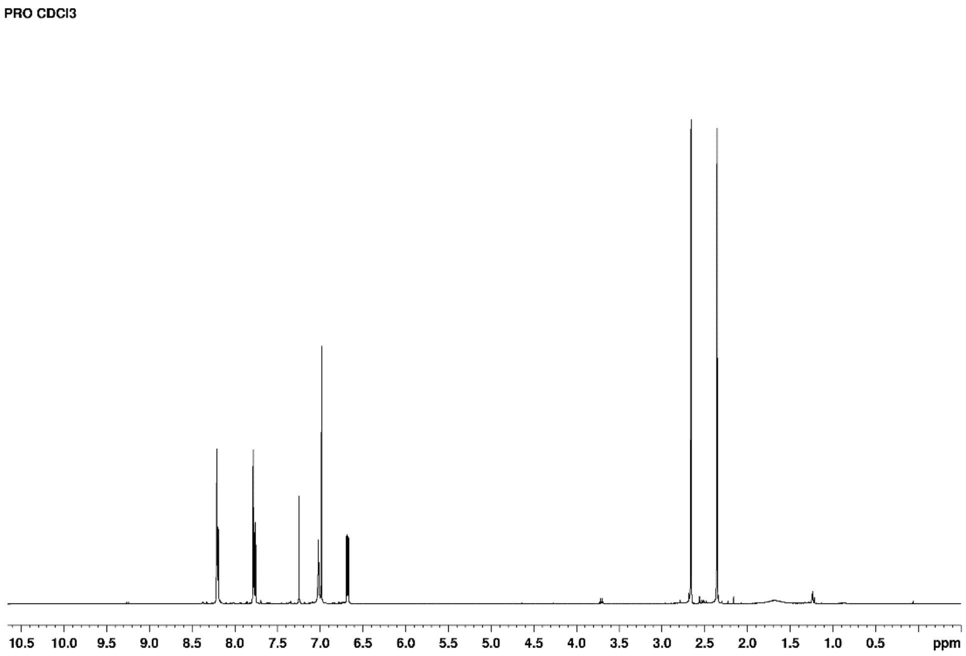
PRO CDCl3
10.5
10.0
9.5
9.0
8.5
8.0
7.5
7.0
6.5
6.0
5.5
5.0
4.5
4.0
3.5
3.0
2.5
2.0
1.5
1.0
0.5
ppm

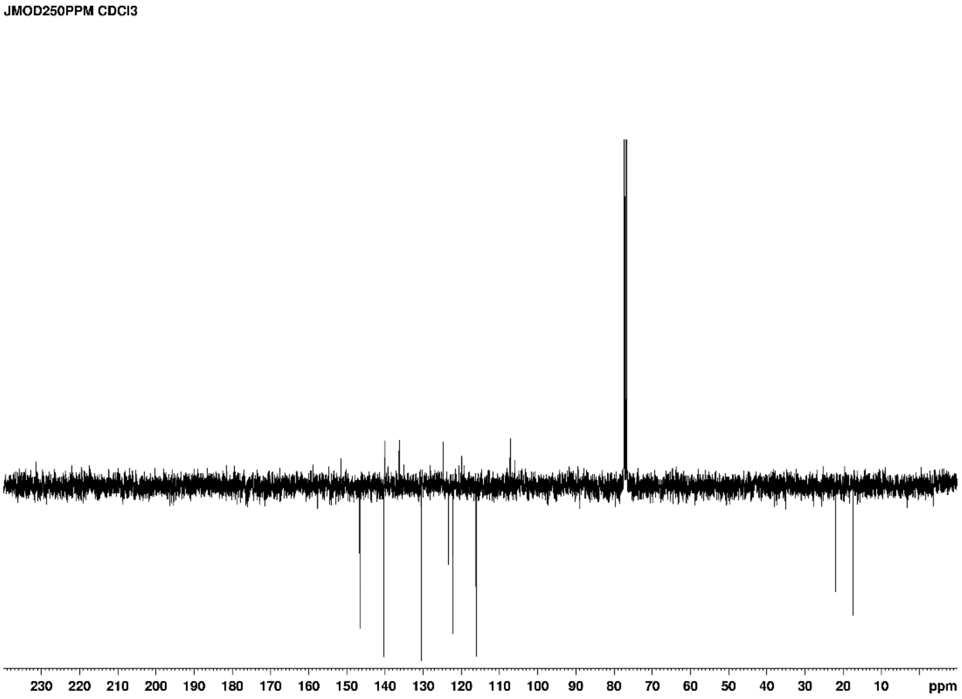
JMOD250PPM CDCl3
230
220
210
200
190
180
170
160
150
140
130
120
110
100
90
80
70
60
50
40
30
20
10
ppm

C13CPD250PPM CDCl3

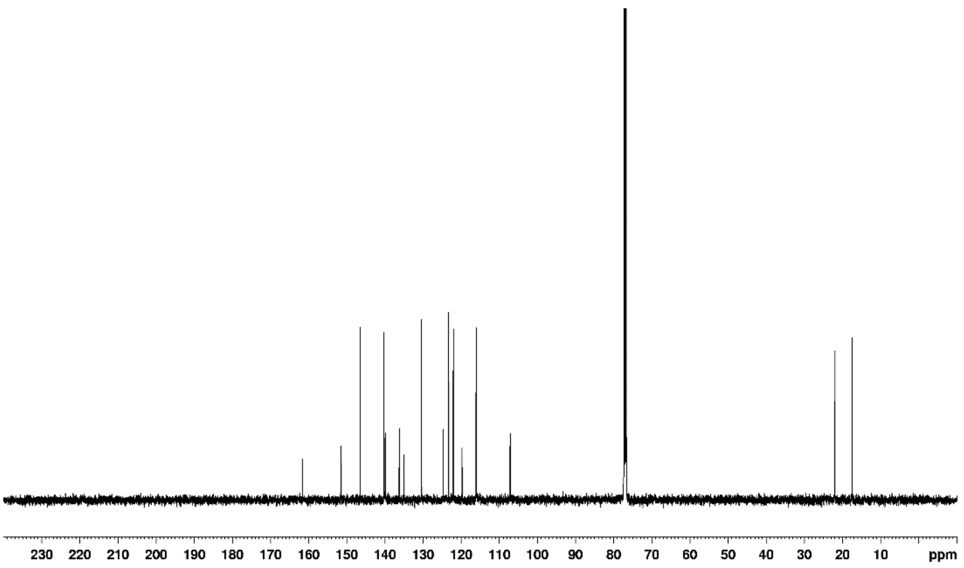

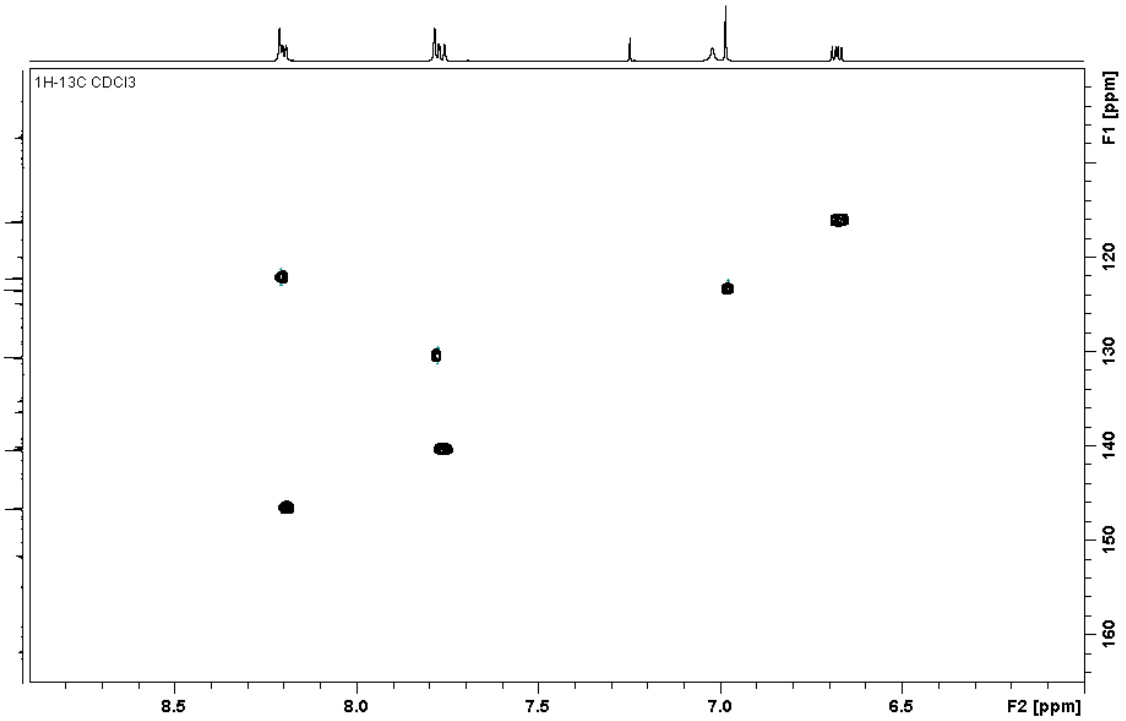

5. Cell biology

Experimental methods

Cell culture, transfection and drug treatments

The plasmids pmCMV-Gl (NORM), pmCMV-Gl (TER) and phCMV-MUP (referred to as NORM, TER and MUP) were a kind gift from Lynne Maquat (University of Rochester, New York). HeLa cells were cultured in DMEM High Glucose (4.5 g/L) with 4 mM L-glutamine (PAA) supplemented with 10% foetal bovine serum (Sigma Aldrich or Gibco) and were incubated at 37 °C with 5% CO_2. Cells were seeded in 6 or 12-well plates at a density of 1–2 x 10^4 cells/cm^2 and incubated for 24 h to allow them to attach to the wells. For transfection experiments, cells were transfected with the MUP plasmid (0.75 µg for 6-well plates or 0.3 µg for 12-well plates) and either the NORM or TER plasmid (0.75 µg for 6-well plates or 0.3 µg for 12-well plates) using Polyfect Transfection Reagent (Qiagen) using the supplied protocol. For 12-well plates, the standard protocol for 6-well plates was scaled down according to the relative surface area of the plates. Cells were exposed to the transfection medium for 24 h. Compound-containing culture medium was prepared with compound at a final concentration of 0.2–125 µM. A 5 mM stock solution of **VG1** or **NMDI1** (a kind gift from Jocelyn Méré, CNRS, Montpelier) in DMSO was used to prepare the medium, with the following exceptions: a 40 mM stock solution in DMSO was used to prepare medium containing **VG1** at a final concentration of 125 or 25 µM and a 0.4 mM stock solution in DMSO was used to prepare medium containing **VG1** at a final concentration of 0.2 µM. The final concentration of DMSO in the medium was adjusted so as to remain constant. Cells were exposed to compound (or DMSO as control) for the indicated times by aspirating untreated culture medium from cells which was replaced with medium containing compound or, as a control, an equivalent volume of DMSO only. Cells were imaged using a Leica DM IL microscope with an AM423X Dino-Eye microscope eyepiece camera. A2780 cis cells were cultured in RPMI1640 supplemented with 10% foetal bovine serum (Sigma Aldrich or Gibco) and treated with 40 µM cisplatin for 36 h prior to lysis and immunoblotting as described below.

RNA analysis

Total RNA was extracted using either Qiagen QIAshredder columns followed by the Qiagen RNeasy kit or by phenol-chloroform extraction using TRI Reagent (Sigma-Aldrich) or TRIzol Reagent (Invitrogen). RNA extraction typically yielded between 1 and 10 µg of RNA (measured using a NanoDrop ND-1000 spectrophotometer). RNA (2 µg per reaction) was reverse transcribed using the Applied Biosystems High Capacity RNA-to-cDNA kit using the supplied protocol. The reaction was incubated at 37 °C for 60 min. The amount of NORM, TER and MUP cDNA was determined by qPCR (where the amount of cDNA serves as a proxy measurement for the amount of mRNA) using a Bio-Rad iCycler thermal cycler with a MyiQ real-time PCR detection system. Master mix containing SYBR Green JumpStart Taq ReadyMix (Sigma-Aldrich, 12.5 µL), forward and reverse primers (Life Technologies, 0.025 µL of a 100 µM stock solution in water) and water (11.45 µL) were added to the wells of a 96-well qPCR plate. cDNA was diluted with water to a concentration of 100 ng/µL, 1 µL of the 100 ng/µL solution was added to the wells and the plate was sealed with StarSeal polyolefin film (STARLAB). The target cDNA was amplified using a program consisting of 40 cycles, utilising a 95 °C dissociation temperature, 60 °C annealing temperature and 72 °C extension temperature. For β-globin (NORM or TER), qPCR primer sequences were 5'-CACCTGGACAACCTCAAGGGCA-3' and 5'-TGCAGCGGGGGTGAAATCCTTG-3'. For MUP, qPCR primer sequences were 5'-CCAATGCCAATCGCTGCCTCCA-3' and 5'-AGGGATGATGGTGGAGTCCTGGTG-3'. Primers were designed using Primer-BLAST online software.[3] The amounts of NORM and TER mRNA, each expressed as a ratio of the respective amount of MUP mRNA in each sample, were compared in order to determine NMD efficiency. Each sample was analysed in triplicate or quadruplicate. Results were quantified by comparison to a standard curve made by combining cDNA from all samples and analysing

serial dilutions on the qPCR plate. A well containing no template was included to check for contamination and the formation of primer dimers. The data were analysed using Microsoft Excel and Bio-Rad CFX Manager. To determine the size of PCR products to confirm that β-globin mRNA generated as a result of transfection was appropriately spliced, RNA derived from cells either mock transfected, or transfected with either pmCMV-Gl (NORM), or pmCMV-Gl (TER) as above, was harvested and reverse transcribed as above, and subjected to standard PCR where each well contained 200 ng total cDNA, 100 pmol each of forward and reverse primers, together with SYBR Green JumpStart Taq ReadyMix (Sigma-Aldrich, 10 μL) in a final volume of 20 μL. β-globin cDNA was amplified using a program consisting of 40 cycles, utilising a 95 °C dissociation temperature, 60 °C annealing temperature and 72 °C extension temperature. DNA electrophoresis was undertaken using 1.5 % agarose gels and reaction products stained with a 0.5 μg/mL solution of ethidium bromide in water prior to imaging using a Uvitech UviProchemi camera system.

Immunoblotting

After indicated treatment, cells were lysed in lysis buffer (20 mM Tris-acetate, pH 7.5, 0.27 M sucrose, 1 mM EDTA, 1 mM EGTA, 1% Triton X-100, 1 mM sodium orthovanadate, 10 mM sodium β-glycoerophosphate, 5 mM sodium pyrophosphate, 50 mM NaF, 0.1% β-mercaptoethanol, 0.2 mM PMSF and EDTA-free protease inhibitor cocktail (Roche). Aliquots of cell lysates were resolved by SDS-PAGE, transferred onto nitrocellulose membrane and probed with the appropriate antibodies. A rabbit polyclonal antibody against cleaved caspase 3 was obtained from Abcam and was used at a 1:500 dilution, and a mouse monoclonal antibody against β-actin was obtained from Sigma-Aldrich, and was used at a 1:20000 dilution. HRP-conjugated anti–rabbit or anti–mouse antibodies (Santa Cruz Biotechnology) were used at a 1:5000 dilution. Blots were visualised using ECL or ECL Prime+ chemiluminescence reagents (GE Healthcare Life Sciences) with X-ray development (Fuji medical film and Optimax 2010 processor).

Data

Figure S1. Cell proliferation and cell death analysis following exposure to VG1. (A) HeLa cells were seeded on tissue culture plates as described and then treated with either DMSO only or 5 µM **VG1** and imaged at indicated times. (B) HeLa cells were seeded on tissue culture plates as in A, treated with DMSO (as a control) or indicated concentrations of **VG1** for 20 h and then imaged. Aberrant cell morphology indicated by white arrowheads. (C) HeLa cells were treated with either DMSO only for 48 h or 10 µM **VG1** for indicated times. Cells were lysed and lysates were subjected to SDS-PAGE and Western blotting. Lysate from A2780 cis cells after 36 h cisplatin treatment was analysed in parallel as a positive control for the immunoblotting experiment. The membrane was probed for cleaved caspase 3 (upper panel) and β-actin (lower panel).

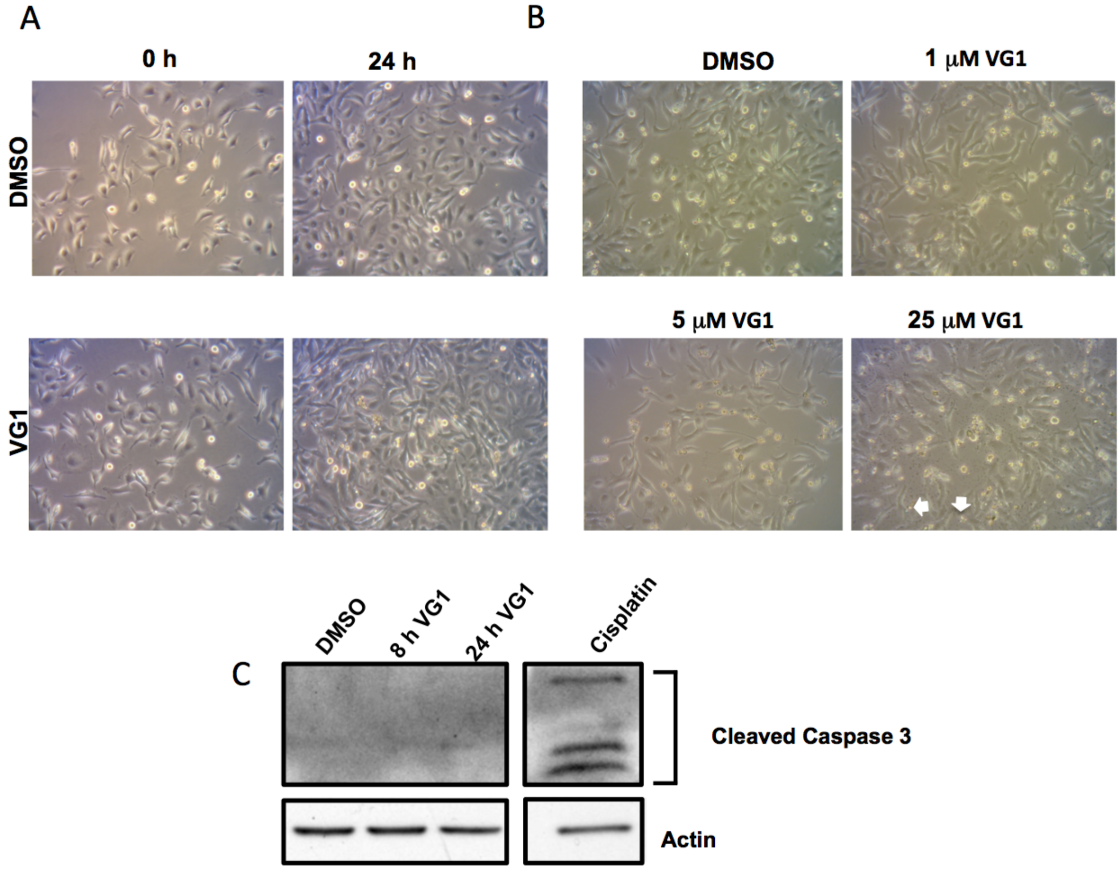

Figure S2. DNA gel electrophoresis of PCR products derived from transcripts of plasmids pmCMV-Gl (NORM), pmCMV-Gl (TER). RNA extracted from untransfected (lanes 1-3) or cells transfected with the indicated plasmid was subjected to reverse transcription and PCR as described above and the size of reaction products determined on 1.5% agarose gels by comparison to DNA molecular weight marker sets (Hyperladder I, upper panels, and Hyperladder V, lower panels (NEB)). The predicted size of the amplified DNA assuming correct splicing is 150bp, whereas unspliced product would be 803bp. Lanes 1 and 2 are controls for DNA amplification as a consequence of cross-contamination.

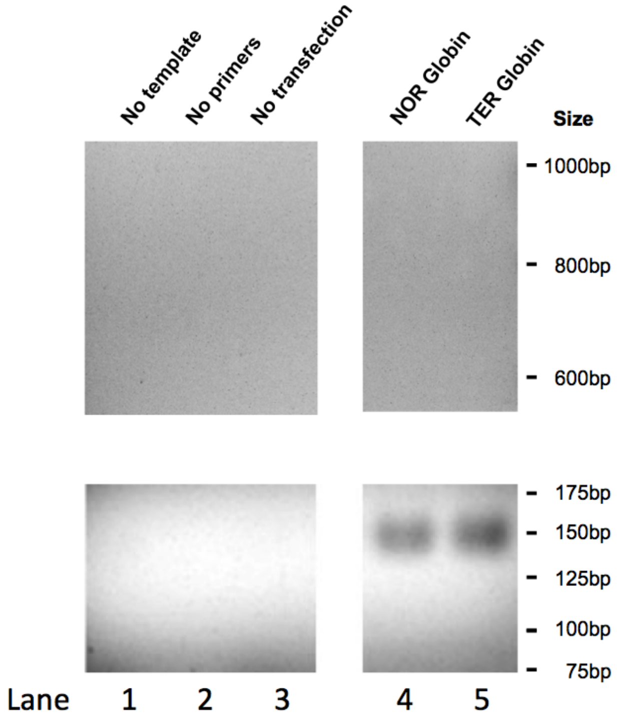

Comparison of NMD inhibition by NMDI1 and VG1 (see Fig. 3A)

Cells transfected with the NMD reporter constructs were treated with either DMSO or 5 µM **NMDI1** or **VG1** for 20 h. The amount of reporter β-globin mRNA (NORM or TER) is controlled to the amount of MUP mRNA. The data are normalised to the amount of NORM mRNA (controlled to the amount of MUP mRNA) for DMSO-treated cells.

	**DMSO**		**NMDI1**		**VG1**	
	**NORM**	**TER**	**NORM**	**TER**	**NORM**	**TER**
Rep 1	1.000	0.226	1.011	0.727	1.111	0.651
Rep 2	1.000	0.233	0.963	0.664	0.980	0.771
Rep 3	1.000	0.205	0.950	0.877	0.928	0.705
**Mean**	**1.000**	**0.221**	**0.974**	**0.756**	**1.006**	**0.709**
**SEM**	**0.000**	**0.009**	**0.018**	**0.063**	**0.055**	**0.035**

Effect of **VG1** dose on NMD inhibition (see Fig. 3B)

Cells transfected with the NMD reporter constructs were treated with **VG1** at the concentrations indicated for 20 h. The amount of TER β-globin mRNA is expressed relative to the amount of NORM β-globin mRNA (both controlled to the amount of MUP mRNA) for each concentration.

	**VG1 concentration (μM)**					
	**0**	**0.2**	**1**	**5**	**25**	**125**
Rep 1	0.301758	0.37587	0.649091	0.76581	0.916192	1.126621
Rep 2	0.228761	0.331138	0.531092	0.708438	0.969982	0.800478
Rep 3	0.195511	0.262181	0.572597	0.856993		
**Mean**	**0.24201**	**0.323063**	**0.58426**	**0.77708**	**0.943087**	**0.96355**
**SEM**	**0.031378**	**0.033067**	**0.034559**	**0.043253**	**0.026895**	**0.163072**

6. References

1. C. Rivalle, C. Ducrocq, J.-M. Lhoste and E. Bisagni, *J. Org. Chem.*, 1980, **45**, 2176.
2. C. Rivalle, C. Ducrocq, J.-M. Lhoste, F. Wendling, F., E. Bisagni, E. and J.-C. Cherman, *Tetrahedron*, 1981, **37**, 2097.
3. http://www.ncbi.nlm.nih.gov/tools/primer-blast.
